# Flying Together: *Drosophila* as a Tool to Understand the Genetics of Human Alcoholism

**DOI:** 10.3390/ijms21186649

**Published:** 2020-09-11

**Authors:** Daniel R. Lathen, Collin B. Merrill, Adrian Rothenfluh

**Affiliations:** 1 Department of Psychiatry and Neuroscience Ph.D. Program, University of Utah, Salt Lake City, UT 84108, USA; d.lathen@utah.edu; 2 Molecular Medicine Program, University of Utah, Salt Lake City, UT 84112, USA; collin.merrill@utah.edu; 3 Department of Neurobiology and Anatomy, University of Utah, Salt Lake City, UT 84132, USA; 4 Department of Human Genetics, University of Utah, Salt Lake City, UT 84112, USA

**Keywords:** genetics, gene discovery, alcohol behavior, *Drosophila*, fruit fly, AUD, alcohol abuse, human, addiction

## Abstract

Alcohol use disorder (AUD) exacts an immense toll on individuals, families, and society. Genetic factors determine up to 60% of an individual’s risk of developing problematic alcohol habits. Effective AUD prevention and treatment requires knowledge of the genes that predispose people to alcoholism, play a role in alcohol responses, and/or contribute to the development of addiction. As a highly tractable and translatable genetic and behavioral model organism, *Drosophila melanogaster* has proven valuable to uncover important genes and mechanistic pathways that have obvious orthologs in humans and that help explain the complexities of addiction. Vinegar flies exhibit remarkably strong face and mechanistic validity as a model for AUDs, permitting many advancements in the quest to understand human genetic involvement in this disease. These advancements occur via approaches that essentially fall into one of two categories: (1) discovering candidate genes via human genome-wide association studies (GWAS), transcriptomics on post-mortem tissue from AUD patients, or relevant physiological connections, then using reverse genetics in flies to validate candidate genes’ roles and investigate their molecular function in the context of alcohol. (2) Utilizing flies to discover candidate genes through unbiased screens, GWAS, quantitative trait locus analyses, transcriptomics, or single-gene studies, then validating their translational role in human genetic surveys. In this review, we highlight the utility of *Drosophila* as a model for alcoholism by surveying recent advances in our understanding of human AUDs that resulted from these various approaches. We summarize the genes that are conserved in alcohol-related function between humans and flies. We also provide insight into some advantages and limitations of these approaches. Overall, this review demonstrates how *Drosophila* have and can be used to answer important genetic questions about alcohol addiction.

## 1. Introduction

Alcohol use disorder (AUD) frequently causes harmful domestic and societal consequences. Alcohol is the most commonly abused drug, and alcohol misuse and abuse are leading causes of preventable death [1,2], underlying ~5.9% of global deaths in 2012 [3]. Additionally, alcohol abuse cost the U.S. ~$249 billion in 2010 [4]. In the U.S. alone, ~18 million people (~7%) have some form of AUD [1], which is defined by the Diagnostic and Statistical Manual of Mental Disorders (DSM-V) as problematic alcohol consumption and use involving craving, dependence, tolerance, withdrawal, relapse, poor decision making, and/or continued consumption despite negative consequences [5].

Genetic factors determine up to 60% of an individual’s risk of developing problematic alcohol habits [6]. Understanding these genetic determinants of AUD risk and associated alcohol responses is critical to predict or alter the propensity for developing AUDs and/or to ameliorate ongoing addiction. This goal is hampered by numerous obstacles: (1) Unlike many drugs of abuse, alcohol does not directly act on one single molecule or system [7,8]. Instead, its targets are diverse and poorly defined. Physiological and behavioral effects of alcohol consumption result from adjustments to a complex assortment of primary targets and their downstream effectors. (2) Increasing evidence suggests that alcohol exerts its effects partially via expression changes of coordinated gene networks rather than of just a few isolated genes [9,10,11,12]. In fact, genetic risk for AUDs appears to result from simultaneous variation of many genes [13,14,15,16], leading to recent shifts away from single-gene models and towards polygenic systems, including complex genetic interactions [17,18,19]. (3) Addiction is a combination of the aforementioned distinct but overlapping behavioral changes, which may have distinct polygenic etiologies. (4) Many influential genes exhibit strong pleiotropy, further complicating the search for genetic determinants. (5) In human genetic studies, it is often difficult to disentangle the role of environmental cofactors in determining AUD phenotypes. (6) Most human genetic studies are correlative by necessity, making it hard to differentiate cause and effects. For example, it is unclear if phenotypes such as altered gross and cellular structure and gene expression in the brains of AUD patients contribute to AUDs or are merely a byproduct of their existence [20,21,22,23]. (7) Human genomic analyses often yield candidate gene sets that share remarkably little overlap with similar human studies [24,25,26]. Even when unambiguous candidates are established, their biological significance is often unclear. Overall, this complexity explains why after decades of research, many of the genetic underpinnings of AUD remain poorly understood.

Model organisms like *Drosophila melanogaster* effectively combat these issues by permitting whole-genome observation of gene expression changes in response to alcohol (e.g., transcriptomics) and, most importantly, by permitting deliberate, reproducible genetic manipulations with minimal environmental confounds. Thus, it is critical to employ animal AUD models to corroborate gene importance and uncover relevant mechanisms. These aims are accomplished by various approaches that generally fall into two categories. The first set starts with gene discovery in humans, usually involving genome-wide association studies (GWAS) or transcriptomics on post-mortem tissue from AUD patients. Candidate genes are subsequently tested via genetic manipulation using the extremely versatile toolkit of *Drosophila* knockouts and transgenes, which are publicly available or inexpensively generated. The second set of approaches works in the opposite direction, where initial gene discovery is made using *Drosophila* forward or reverse genetic screens, GWAS or quantitative trait locus (QTL) analyses, or transcriptomics after alcohol exposure. Candidates are then compared to human forward genetics data or used to guide hypothesis-driven reverse genetics studies, such as candidate gene association studies (CGAS). These complementary methods contribute substantially to our knowledge of the genes that predispose individuals to AUDs and/or contribute to AUD development and maintenance. Together, these approaches create a rich knowledge base upon which further investigations can be founded.

Here, we summarize key attributes of *Drosophila melanogaster* that make this model organism especially useful for AUD research. We then explain approaches used in humans and flies to discover, validate, and investigate candidate genes, summarize important findings contributed by each approach, and discuss their strengths and weaknesses.

## 2. *Drosophila Melanogaster* Is a Tractable Model for AUD

### 2.1. Advantages of Using Flies for AUD Research

Approximately one-hundred genes have been implicated in AUDs by correlation in human genetic studies, while thousands more have emerged from human transcriptomic approaches. Although such nominal association could prove useful in estimating predisposition to develop AUDs, the magnitude of these numbers prevents effective hypothesis-driven research into whether or not these genes causally contribute to AUDs and, if so, the underlying mechanisms. To address this problem, the pool of potential AUD genes must be qualitatively filtered. Furthermore, ethical considerations prevent detailed genetic analyses in humans, so genetically tractable model systems are required. Many attributes of *Drosophila* make them an effective model for alcoholism studies (Note: out of all *Drosophila* species, only *Drosophila melanogaster* has been studied extensively in the context of alcoholism. Hereafter, “*Drosophila*” and “fly” will refer exclusively to *Drosophila melanogaster*). *Drosophila* solves the aforementioned problems by (1) permitting efficient reverse genetics hypothesis testing of genes linked to AUDs in other model systems and (2) acting as a tractable platform for discovery of alcohol-related genes and gene networks that can subsequently be tested in human studies.

Most mammalian genes have orthologs in flies [27], including all major human gene families [28]. More specifically, an estimated 75% of human disease genes have known *Drosophila* orthologs [29], providing strong evidence that this system has substantial worth as a platform for discovery of conserved genes and elucidation of mechanisms relevant to AUDs. (Many genes linked to AUD phenotypes in both humans and flies are summarized in Table 1, some of which are discussed in greater detail below). Findings from *Drosophila* have high translational value. Homologous genes that affect phenotypes represent valuable targets for further mechanistic studies and potential therapeutic targets. Furthermore, fly gene families often have fewer members and less redundancy than those of mammals [27,30,31]. This fact simplifies forward and reverse genetic approaches and makes it more likely that epistatic experiments can reveal the members and orders of genetic pathways. Flies also possess a rapid life cycle and high fecundity, which permit economical husbandry, efficient gene discovery, and mechanistic experiments with high statistical power. Indeed, researchers can perform high-throughput genomic studies and forward screens in flies at only a fraction of the cost and time required for equivalent rodent or human experiments.

The well-established *Drosophila* research community has generated a myriad of easily obtainable genetic resources, comprising the largest collection of readily available transgenes and other genetic tools. Including RNAi-lines for gene knockdown, easily obtainable mutant strains exist for the majority of fly genes, whether created by CRISPR/Cas9, homologous recombination, or more classic methods [111]. These tools enable efficient hypothesis testing and complex, precise genetic manipulations that are important for validation and elucidation of genes implicated in unbiased studies. For example, Morozova et al. selected 37 candidate gene mutations from a transcriptional comparison of ethanol-sensitive versus -resistant fly strains and showed altered sensitivity to sedation in 32 of them [80].

**Figure 1:**
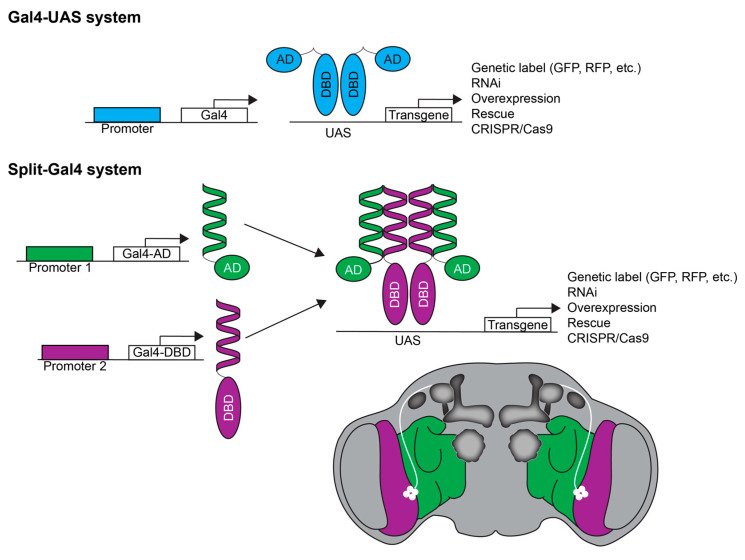
The Gal4-UAS system allows precise control of transgene expression. In this binary system, the yeast transcription factor Gal4 is placed under the control of a specific gene promoter, which limits Gal4 expression to select cell types expressing the driver gene. This transgenic construct is combined with a second transgene that places a desired effector gene downstream of the Gal4-binding upstream activation sequence (UAS). Thus, the expression of the effector gene is under spatial and temporal control of a specific gene promoter. The split-Gal4 system uses an intersectional approach to refine Gal4 expression. The Gal4 activation domain (AD) and DNA-binding domain (DBD) are placed downstream of two different promoters. In cells that express both promoters, the AD and DBD combine to form a functional Gal4 protein, which then binds the UAS and drives transgene expression in a more spatially restricted manner. For example, in brain areas where AD (green region) and DBD (purple region) expression overlap (white neurons), the UAS is expressed.

Among the most important genetic tools is the Gal4/UAS system, which permits complex, cell-specific genetic manipulations such as genetic labeling, overexpression, RNAi-mediated transcript knockdown, gene rescue, and diverse CRISPR/Cas9-mediated gene edition [112] (Figure 1). Each of these outcomes can be limited to specific developmental time points, cell populations, or both. Moreover, temperature-sensitive or light-regulated effector genes can silence or activate neurons expressing a gene of interest in a temporally restricted manner. Massive collaborative projects have resulted in Gal4/UAS tools becoming available for most known fly genes. RNAi transgenes are available for almost any gene of interest, and thousands of Gal4 drivers are available that drive expression in distinct subsets of neurons [113]. Understanding the anatomical specificity of addiction genes is important, given that the same genetic manipulation can cause differing results depending on the precise locus of action. For example, global expression of a protein kinase A (PKA) inhibitor causes sensitivity to alcohol sedation [114], while anatomically limited inhibition causes resistance or sensitivity, depending on the neuroanatomical locus [65,114]. Many studies demonstrate the utility of these *Drosophila* genetic tools to establish causal roles of various genes in alcohol phenotypes, including many linked to specific cell populations [31,66,101,115,116,117,118,119,120,121]. The split-Gal4 system permits even further refinement by limiting manipulations to subsets defined by two criteria (e.g., GABAergic neurons in the ellipsoid body) [122], thus allowing investigation into the contribution of neuronal subpopulations or even individual neurons to the development of alcohol abuse disorders (Figure 1). Using such tools, specific neuronal populations are easily targeted in flies via straightforward crosses, rather than relatively difficult virally mediated targeting in mammals. These neuronal subsets may include neurotransmitter systems, which are highly conserved, and/or specific brain regions, which, while not structurally homologous between humans and flies, are often analogous in function. The Gal4/UAS system also allows expression of fluorescent proteins or tagged proteins limited to specific cell types of interest. This advantage permits cell-type-specific visualization, sorting, and transcript analyses using assays such as isolation of nuclei tagged in specific cell types (INTACT) [123,124], translating ribosome affinity purification (TRAP) [125], and chromatin affinity purification (CAST-ChIP) [126] (see also Reference [127] for review).

Genome-wide transcription analyses (transcriptomics), which are already readily performed in flies, can become even more refined using these tools. As additional omics methods, assay for transposase-accessible chromatin-sequencing (ATAC-seq) and chromatin immunoprecipitation sequencing (ChIP-seq) can be performed in flies. These assays represent effective ways to investigate the genome-wide effects of alcohol exposure on chromatin remodeling and DNA binding of proteins such as transcription factors and epigenetic enzymes, respectively. Performing these tests with human tissue is rare and impossible to perform after controlled alcohol exposure or to restrict to specific cell types, though isolating specific brain regions is feasible. In flies, but not mammals, genes identified from ATAC-seq or other omics methods can be easily integrated into transgenes and functionally tested [128]. Similarly, important human SNPs or human orthologs of genes of interest can be engineered in flies to explore their biochemical or behavioral roles [129,130,131]. Finally, despite their relatively simple nervous systems, flies retain a fairly complex behavioral repertoire that mirrors many behavioral paradigms found in vertebrate models, again demonstrating their usefulness in AUD research [111].

### 2.2. Drosophila Alcohol Assays Establish Flies as an Effective AUD Model System

Since addiction is a complex combination of various behaviors, researchers generally break down AUDs into discrete aspects of addiction represented by specific behavioral responses (i.e., endophenotypes), such as naïve ethanol (EtOH) sensitivity, functional tolerance (brain-mediated decreases in response resulting from repeated exposures), or alcohol consumption. Many of these distinct behaviors can act as metrics to indicate human propensity for developing AUDs. Specifically, AUD risk is augmented in individuals exhibiting reduced alcohol sensitivity, greater tolerance, increased consumption, greater stress, and greater EtOH dependence [132,133,134,135,136]. *Drosophila* are useful for uncovering the genetic underpinnings of these endophenotypes because many of these important response metrics can be modeled and reproducibly quantitated in fly behavioral assays. In fact, the validity of this model system has been established in parallel with development of various innovative assays that permit research into *Drosophila* EtOH responses and addiction. Partly due to the substantial similarities between human and fly AUD phenotypes (i.e., strong face validity), it is now widely accepted that flies are a powerful model for alcohol abuse [99,101,111,137,138,139].

**Figure 2:**
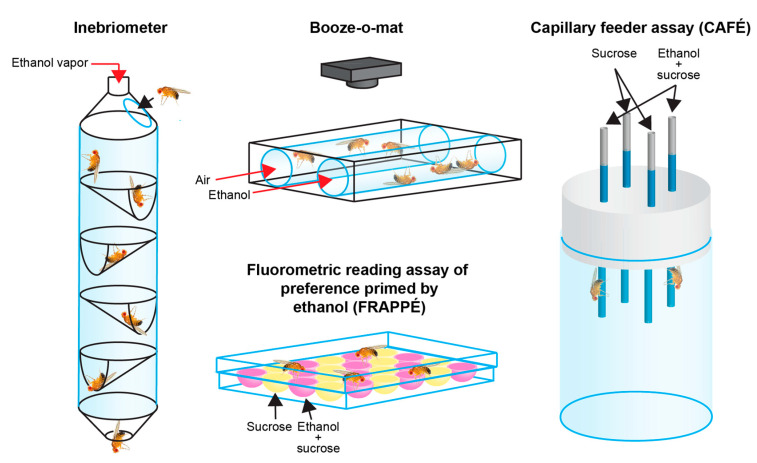
Assays used to test alcohol-related behaviors in *Drosophila*. The inebriometer measures sensitivity as a function of loss of postural control by determining the amount of time required for EtOH-exposed flies to “elute” out of a column with interspaced oblique baffles. The “Booze-o-mat” assay employs video tracking of fly postural control and/or movement during vaporized EtOH exposure to determine flies’ naïve alcohol sensitivity. Consumption assays such as the capillary feeder (CAFÉ) and the fluorometric reading assay of preference primed by ethanol (FRAPPÉ) determine flies’ preference for EtOH-containing food compared to control solutions. Different consumption assays permit different temporal resolution.

Like humans, flies become hyperactive and disinhibited upon exposure to low doses of EtOH, uncoordinated at moderate doses, and sedated at high doses [114,140,141,142]. The original test to quantify EtOH sedation was the fly “inebriometer” [143] (Figure 2). More recent assays, such as the “Booze-o-mat,” determine flies’ naïve sensitivity to alcohol’s effects by providing measurements of hyperactivity, postural control, sedation, and time to recovery after EtOH cessation [111]. These tests also show that flies develop rapid tolerance (i.e., they require longer to sedate upon second exposure after all EtOH from initial exposure has completely metabolized) [46,144]. Rapid tolerance forms in as little as two hours and can persist for 24 h [46] or for weeks, depending on methodology and genotype [145]. Importantly, fly tolerance studies consistently find that EtOH absorption and metabolism do not change between first and subsequent alcohol exposures, nor between treatment groups with differing sensitivity or tolerance [46,69,144]. Thus, observed differences in sedation upon repeat exposure result from functional tolerance (mediated by the nervous system), not metabolic tolerance (mediated by altered activity of enzymes that metabolize EtOH). Given that alcohol addiction in humans largely depends on the development of functional tolerance, this fact again demonstrates the usefulness of adult flies to study AUDs.

Flies also develop symptoms of alcohol dependence and withdrawal. For instance, similar to humans, larvae experience neuronal hyperexcitability resulting from EtOH withdrawal, a finding that holds true for adult flies [70,71,146]. Further, fly larvae exhibit decreased learning ability during withdrawal compared to unexposed and re-inebriated flies, indicating cognitive dependence [147].

Last, various preference assays have been utilized to discover important similarities between fly and human EtOH preference responses that help to establish face validity of this model organism. Kaun and colleagues found robust preference learning by employing an odor-pairing Y-maze, demonstrating that, as in humans, alcohol acts as a behavioral reinforcer in flies, similar to analogous findings in rodents using conditioned place preference tests [148]. Consumption studies using assays such as the capillary feeder (CAFÉ), the fluorometric reading assay of preference primed by ethanol (FRAPPÉ), and the proboscis extension reflex (PER) reveal that, like humans, naïve flies are initially indifferent toward or avoidant of alcohol, depending on exposure method and parameters [149,150] (Figure 2). However, after prior alcohol experience, they develop EtOH preference, which increases over time and rebounds strongly after a period of abstinence [149], reminiscent of increasing intake and relapse in human AUD patients. In conjunction with aversive stimuli such as quinine (bitter taste) or electrical shock, these assays also show that flies will overcome negative stimuli in order to self-administer [148,149]. It should be noted that consumption tests often involve prior starvation, which may cause confounding effects via activation of stress pathways and genetic networks unrelated to alcohol responses [151]. Nonetheless, these and similar assays are frequently used to effectively quantify alcohol preference in flies. Overall, these various translatable assays enable robust and rapid functional testing of genes nominally implicated in AUDs.

## 3. From Mammalian Gene Discovery to Fly Functional Testing

Human and rodent studies have successfully utilized various approaches to identify many genes associated with AUDs. These approaches are discussed below, including GWAS, post-mortem transcriptomics, QTL analyses, and investigation of genes with known physiological connections. Functional testing to verify the roles of these genes is important. Therapeutic targeting of suspected AUD genes is more likely to be safe and effective if their mechanistic underpinnings are well understood. Some genes consistently associated with alcoholism in humans have clear mechanisms, such as the EtOH metabolism gene aldehyde dehydrogenase 2 (*ALDH2*). ALDH enzymes break down acetaldehyde, a molecule which causes nausea, facial flushing, and tachycardia. People with less efficient *ALDH2* alleles experience more severe reactions, are more likely to be deterred by this aversive reaction, and are thus less likely to develop AUDs [152,153]. Contrastingly, some genes such as *AUTS2* (discussed in detail below) are frequently implicated in human studies, yet have poorly understood function and no established physiological links to AUD phenotypes [38,39,40]. Thus, functional and mechanistic studies in model organisms are crucial. Building off of human gene discovery, *Drosophila* can be used to reveal the roles of functional protein states such as expression levels, localization, post-translational modifications, binding to other proteins or nucleic acids, etc. Given the wealth of available tools and assays, *Drosophila* represent an efficient and effective way to test roles and mechanisms of potential genes contributing to AUD risk, formation, and maintenance. The candidate genes that fuel such functional studies in flies arise from several different approaches. Below, we discuss each approach, including advantages, limitations, and examples of studies that have applied it to uncover candidate genes that were later successfully validated in flies.

### 3.1. Human Genome-Wide Association Studies (GWAS)

GWAS have revealed a substantial number of candidate AUD genes. This approach finds associations between inherent DNA sequence polymorphisms (or sets of polymorphisms) and AUD phenotypes measured by alcohol consumption, dependence, maximum drinks over a given time span, etc. Currently, most individuals at high risk for AUD discover their heightened genetic risk factors only after they develop a problem, if ever. In contrast, combined with the ever-increasing ease of full-genome sequencing, genetic players found using GWA analyses can potentially reveal high inherited susceptibility for AUD before the disease happens. Given that genetic propensity for AUD is extremely heterogeneous [19], treatment considerations for extant pathologies may also be guided in the future by understanding individuals’ particular genetic backgrounds.

Many important discoveries have been made using the GWAS approach. One gene implicated in multiple GWAS is *AUTS2*, a nuclear protein that interacts with polycomb repressor complexes, which play a role in gene regulation via chromatin remodeling [154]. Schumann et al. identified this locus via GWA meta-analyses using alcohol consumption as the dependent variable [40]. They then found increased *AUTS2* expression in the human prefrontal cortex from carriers of a minor *AUTS2* allele, as well as altered expression between various high alcohol-preferring and low-alcohol-preferring mouse lines. The human importance of *AUTS2* is further supported by another GWAS of alcohol consumption, a GWA meta-analysis of the maximum number of drinks consumed in 24 h, and a biased haplotype analysis [38,39,155]. Lastly, Schumann et al. showed that reduced expression of the fly ortholog *tay* reduces EtOH sensitivity. *tay* negatively regulates the epidermal growth factor receptor (EGFR) pathway [156]. EGFR signaling plays diverse roles in flies [157], especially during development, is responsive to promising, FDA-approved drugs in humans, and is frequently implicated in fly alcohol behavior [51,80,82,158,159,160]. For instance, EGFR suppresses EtOH-induced locomotion [159]. Thus, *tay* and *AUTS2* may affect alcohol behaviors through this pathway.

As another example of genes elucidated using GWAS, Schmitt and colleagues performed a meta-analysis of GWAS data on the endophenotype known as SRE (Self-Rating of the Effects of alcohol), yielding 37 hits, including the transcription factor *MEF2B* [75]. Follow-up validation of *Drosophila* orthologs revealed that loss-of-function mutations of the *Mef2* transcription factor decrease EtOH sedation sensitivity but not rapid tolerance. Another group found that Mef2 reduction in neurons, or more specifically in mushroom body α/β neurons, reduces tolerance, corroborating the importance of this gene in alcohol responses [78]. The dissimilar fly tolerance results between these two groups may be an example of global gene manipulations causing different effects than manipulations limited to particular neuronal populations. A recent CGAS by Muench et al., and a human GWAS meta-analysis by Evangelou et al., further corroborate the role of *Mef2* by implicating the human ortholog *MEF2C* [76,77]. Though the exact mechanisms of action remain unclear, mammalian *Mef2A* and *Mef2D* regulate dendrite differentiation and synapse number [161,162]. Further, signaling pathways affected by EtOH control *Mef2* expression and activity, as do pathways linked to neural activity (e.g., intracellular calcium) [161,163,164]. Indeed, Sivachenko and colleagues showed a role for *Mef2* in fly neuronal plasticity, including temporal cycling of neuronal cytoskeleton structure, suggesting intriguing connections to adaptive neuronal processes involved in addiction [165]. Supporting these hypotheses, other work shows that *Mef2* suppresses cocaine-induced increases in dendritic spine density [166]. Finally, Adkins et al. found that the *RYR3* gene, encoding a ryanodine receptor regulating intracellular calcium levels, has a “suggestive association” with human alcohol dependence [102]. This finding was not significant in replication; however, loss of the fly ortholog *RYR* notably reduced rapid tolerance to EtOH-induced sedation, highlighting the importance of functional validation of findings that may appear inconsistent in human studies due to limited sample sizes and low statistical power.

A brief discussion of caveats to GWAS studies is warranted. For instance, given that increasing evidence supports a role of epigenetics in mediating EtOH responses, it is important to note that there are potential disconnects between the genomic sequences studied in GWAS and the true transcriptional states that contribute to EtOH phenotypes and AUD susceptibility. Additionally, genes implicated in GWAS may not in and of themselves produce acute EtOH responses or the adaptations that lead to addiction. Candidate genes could be upstream regulators of the actual effector genes, including regulators involved in distinct but relevant processes such as executive function, motivation, and decision-making. Historically, GWAS can also suffer from selection bias, environmental confounds, poor reproducibility, and weak statistical power, largely due to vast heterogeneity between subjects and studies. Although some GWAS studies yield very few or no genetic variants that reach genome-wide significance, these shortcomings are increasingly ameliorated by increased sample sizes, pooled meta-analyses, and improved “post-GWAS” methods [24,25]. For instance, Evangelou et al. performed a meta-analysis of GWAS data of alcohol consumption from almost 500,000 people, which had enough power to identify 46 putative AUD genes [76]. Indeed, with this success comes the problem of functionally validating so many potential hits, given that they found no overrepresentation of cohesive gene families, pathways, or ontologies. This challenge becomes manageable by turning to high-throughput models like *Drosophila* for gene validation. Similarly, for gene discovery, many concerns of human GWAS are diminished in fly GWAS and other fly studies, which are amenable to higher sample number and greater statistical power (discussed below).

### 3.2. Transcriptomics on Post-Mortem Human Tissue

One approach to connect gene expression to psychiatric disease is the application of transcriptomics to brain tissue from deceased AUD patients versus healthy controls [10,12,20,23]. These approaches uncover associations between the severity of AUD phenotypes and global or region-specific gene expression. Moreover, they often use network analyses to distinguish genes that are expressly altered by AUDs from genes that may be dysregulated merely as part of co-regulated EtOH-responsive networks. Transcriptome profiling is generally accomplished with microarrays or RNA-sequencing (RNA-seq). Whole-genome profiling using these methods demonstrates the effects of chronic alcohol use on gene expression in various brain regions known to play a role in AUDs, including the prefrontal cortex, nucleus accumbens, and hippocampus [12,20,167,168,169]. Building upon existing human transcriptome data from microarrays [41], one group performed gene set overlap analysis between this data, transcriptomics on mice exposed to EtOH, and transcriptomics comparing isogenic mice bred to be alcohol-preferring or non-preferring [42]. Using this combined approach, they identified the most highly ranked hit, a chloride intracellular channel known as *Clic4*, as a potential AUD gene. Subsequent validation in flies (mutants), *C. elegans* (mutants), and mice (virally mediated overexpression) showed significantly altered alcohol sensitivity. EtOH sensitivity in loss-of-function *Drosophila* mutants was consistently decreased across studies, despite dissimilar assay methods [42,43], and also in flies with neuron-specific RNAi knockdown of *Clic4* [43].

One weakness of transcriptomic approaches is that they establish only correlational links between genetic state and disease phenotypes. Additional weaknesses include the challenges of RNA degradation, heterogeneity between individuals, environmental confounds, and highly dynamic transcriptional adaptation in response to unpredictable stimuli. Hence, highly controlled functional testing in flies is all the more critical.

As a notable alternative to typical transcriptomics, two groups performed ChIP-seq on post-mortem samples to show that, like gene expression, histone methylation is altered in the brains of alcoholics [10,169]. These studies supported later fly research that revealed a role of various histone demethylases in alcohol responses [121]. Given that covalent epigenetic markers are more stable than mRNA, there is great potential for epigenome studies in this context, though these approaches are still in their infancy [10]. Nonetheless, these omics methods provide in-depth genetic profiles separable by brain region and remain as powerful tools to directly study AUDs in humans.

### 3.3. Rodent GWAS, QTL Analyses, and Transcriptomics

As an alternative to the human approaches already discussed, important gene discovery can also be accomplished with rodent models. These approaches may include similar post-mortem transcriptomics and GWAS-style analyses, with the additional possibility of performing QTL analysis on rodent lines with purposefully limited genetic variation [170]. QTL studies identify genomic regions whose genetic variation or expression correlate with quantification of phenotypes of interest. Investigation of rodent gene expression profiles after EtOH exposure can also yield useful information, similar to flies (see below). Methods to study AUD genetics in rodents have been reviewed extensively and will not be discussed in-depth here [171,172]. However, one noteworthy example is a study by Mulligan et al. This group demonstrated the effectiveness of meta-analysis combining rodent genetics (using congenic strains) and transcriptomics (using microarray after alcohol exposure) [173]. In their results, they highlighted *EGFR* signaling and cytoskeleton regulation as some of the most overrepresented pathways differentially expressed between mice stains exhibiting differential alcohol consumption, converging with the aforementioned *AUTS2*/*EGFR* studies and with findings implicating cytoskeleton dynamics using forward genetics in flies (see below).

Additionally, the neuropeptide NPY (fly ortholog: NPF) and its receptors are notable examples of many effective rodent methods, which were subsequently applied to yield corresponding fly and human discoveries that revealed the mechanistic conservation of this gene in AUDs (for review, see Reference [174]). *NPY*/*NPF* controls both hunger and stress levels. This gene was initially implicated by a QTL analysis and comparisons of expression levels between alcohol-preferring versus non-preferring rats, and by measuring *NPY* transcript levels in wild-type rats with or without EtOH exposure [170,175]. Thiele and colleagues also found that NPY deficiency in mice increases EtOH consumption and resistance, while overexpression reduces these phenotypes [176]. In flies, NPF modulates reward states [95], confirmed recently in a study that used optogenetics to allow flies to self-administer by moving to the appropriate area, then tested the flies’ conditioned place preference [177]. Similar to rodents, reduction of NPF (or its receptor, NPFR) increases EtOH resistance, while overexpression has the opposite effect [93]. Sekhon et al. also tested inbred fly lines to associate *NPF* and *NPFR* with altered EtOH preference [52]. Completing the picture, *NPY* and *NPY* receptors have been implicated in numerous human studies [36,88,89,90,91,92,94]. Work on *NPF*/*NPY* exemplifies a primary strength of rodent gene discovery: greater cross-species validation strengthens confidence that the gene is causally involved in AUDs. If a gene discovered in rodents can be successfully validated and mechanistically explored in flies and demonstrated to associate with AUD in humans, such a conserved role despite vast evolutionary distances strongly suggests a role for the gene in AUDs and great promise as a potential therapeutic target.

### 3.4. Targeting Genes with Established Physiological Relevance

In addition to the approaches discussed so far, researchers also perform functional testing of genes in flies in response to prior association with relevant gene networks or physiological processes known to play a role in rodent or human AUDs, independent of any large-scale omics or GWAS studies. Genes investigated for this reason include various synthesis enzymes, transporters, receptors, and degradation enzymes for neurotransmitters such as dopamine, serotonin, GABA, glutamate, and octopamine [6,111,138,148,178]. Octopamine is the functional equivalent of norepinephrine in *Drosophila* [179]. Further examples include CREB, CREB binding protein (CBP), and the BK-type Ca^2+^-activated K^+^ channel, *slo* [6,111,138,178,180]. Given the vast collection of literature supporting roles for these genes in AUD, only one will be discussed here. *slo* was first investigated in the context of alcoholism because it undergoes homeostatic regulation after sedation by organic solvents and plays a role in tolerance to benzyl alcohol [138]. Loss of *slo* globally or in neurons eliminates EtOH tolerance [69], while *slo* induction is sufficient to produce a tolerance-like phenotype [70]. Further, EtOH sedation increases *slo* expression in neurons but not in non-neuronal tissue, which is concomitant with tolerance formation [70]. In flies, neuronal hyperexcitability resulting from EtOH withdrawal is at least partially dependent on persistent *slo* upregulation [71,72]. These types of ion channels may play a role in maladaptive brain plasticity leading to AUDs in humans, supporting the mechanistic validity of fly models [181]. Finally, two separate GWAS studies associated the human ortholog *KCNMA1* (potassium calcium-activated channel subfamily M alpha 1) with alcohol dependence [34,68]. Thus, established physiological relevance laid the foundation for mechanistic AUD hypotheses and important discoveries of the role of *slo* in flies and humans.

### 3.5. Summary of Human-to-Fly Approaches

Various approaches permit effective gene discovery in mammalian systems. Though easily translatable, it is often difficult to assess the causative role of candidate genes in observed AUD phenotypes. The examples cited above demonstrate the usefulness of *Drosophila* to accomplish this purpose. Notably, some genes remain implicated in multiple human studies that, to our knowledge, have not yet been examined in *Drosophila*. For instance, β-Klotho (gene name: *KLB*), a transmembrane protein that complexes with fibroblast growth factor receptors (FGFR), was implicated in a human GWAS and a separate GWAS meta-analysis investigating alcohol consumption [38,182]. The latter study by Schumann et al. also found that *KLB* knockout mice have increased alcohol preference. Although King et al. showed that mutations in the fly FGFR gene *htl* reduce EtOH-induced locomotion [159], further validation of the role of *KLB* in AUD phenotypes is still needed, as is greater mechanistic understanding. Investigation of the mostly uncharacterized fly ortholog, *CG9701*, have potential to address these important gaps. Other interesting examples are various genes involved in serotonergic neurotransmission, which have been implicated in both biased and unbiased human genetic studies but have not yet been directly tested in flies [183,184,185]. Serotonin signaling clearly plays a role in alcohol responses, but much mechanistic insight could be gained by using flies to determine the effects of manipulating these various genes in specific neural populations and/or at specific developmental timepoints.

## 4. From Fly Gene Discovery to Human Association

Complementing the approaches already discussed, research can proceed in the opposite direction, wherein AUD gene discovery begins in *Drosophila* and moves to human validation. This overall approach is advantageous because, as a more efficient and genetically tractable animal model, gene discovery occurs faster in flies than in mammals. Human validation takes the form of candidate gene association studies (CGAS), which use reverse genetics to test associations between phenotypes of interest and small numbers of genes hypothesized to be important. Compared to GWAS, CGAS represent a more effective method of investigating specific disease questions. Critically, limiting the pool of candidate genes also limits the problem of multiple comparisons, creating more power for discovery of relevant polymorphisms despite low frequencies, subtle effects, or smaller sample sizes. Overall, since gene discovery in flies is generally accompanied by mechanistic and functional tests, approaching questions in this way combines the *Drosophila* strengths of breadth and depth with the mammalian strength of high translational value.

### 4.1. Behavioral Screens in Drosophila

Gene discovery in flies often begins with large-scale forward screens which remain practical due to the ease of generating random or deliberate mutations and the ease of quickly generating and testing thousands of flies in high-throughput assays. Unbiased forward screens begin with genetic mutagenesis accomplished with chemical agents, radiation, CRISPR/Cas9 [186,187], or transposable elements to establish hundreds of different fly strains. These strains are each scored for a given behavioral readout to detect aberrant phenotypes. Subsequent genetic mapping, DNA-sequencing, and rescue experiments then confirm the identities and causative roles of disrupted genes so that researchers can draw conclusions about their involvement in the phenotypes of interest.

Single gene discoveries made in flies using one method easily expand into elucidation of entire pathways found gene-by-gene using a variety of complementary approaches. Such was the case after Rothenfluh and colleagues performed a transposable P-element screen of ~1200 fly strains, examining EtOH-induced phenotypes [188]. They identified mutations in *RhoGAP18B*, a GTPase-activating protein (GAP). RhoGAP18B binds and inactivates actin-regulating Rho-family GTPases such as Rac1 and Rho1. Accordingly, loss-of-function mutations of *RhoGAP18B* and hyperactive Rac1 or Rho1 cause resistance to EtOH sedation [188,189]. Independently, loss-of-function mutations in *Rsu1*, another Rac1 inhibitor, were also found to cause resistance to alcohol sedation [101]. Hypothesis-driven CGAS in the same study found associations between human *RSU1* polymorphisms and alcohol consumption in two independent cohorts. These initial human findings suggest that this pathway plays a conserved role in alcohol responses and demonstrate the utility of fly gene discovery followed by human hypothesis testing.

Reverse genetics testing of related genes has further expanded the pathway to include upstream and downstream players such as the integrin cell-adhesion molecule and cofilin, an actin-severing protein, respectively [101,189]. Cofilin modulates actin cytoskeleton dynamics, suggesting a mechanism through which these genes could affect neuroplasticity and alcoholism [115]. To identify additional participants in the pathway, a subset of 300 randomly selected mutants was screened for effects on semi-lethality, a distinct pleiotropic phenotype of the strongest *RhoGAP18B* allele [100]. EtOH responses were tested in mutant lines implicated by the first screen. This iterative method identified *Efa6*, a guanine exchange factor (GEF) and activator for the small GTPase Arf6 [99]. Further hypothesis testing of *Efa6* by Gonzalez et al. and Peru et al. found that *Arf6* and *Efa6* mutant flies exhibit increased sedation sensitivity and decreased tolerance [99,100]. Gonzalez et al. further showed that a SNP in one of four human *Efa6* orthologs, *PSD3*, and a haplotype containing this SNP were associated with adolescent binge drinking and frequency of consumption. Moreover, the haplotype was linked to increased dependence in an independent sample. These human studies revealed that *PSD3* expression is mostly restricted to the brain and is especially high in the prefrontal cortex. Of the four human orthologs, *PSD3* exhibits the most limited expression patterns, suggesting less pleiotropy and higher potential for drug targeting. Finally, reverse genetics hypothesis testing elucidated the identity and relative order of various genes connected to *Arf6* that form a pathway parallel to that of *RhoGAP18B*, including insulin receptor (*InR*) upstream and *mTor* and S6 kinase (*S6K*) downstream [66]. Inhibition of the mammalian ortholog *mTORC1* with the FDA-approved drug rapamycin reduces alcohol seeking and drinking in mice [190,191,192]. Overall, this process of gene detection and testing demonstrates how screens and hypothesis-driven testing in flies and humans can work together to discover novel pathways with high potential for targeted drug therapy.

Forward screens were also used by Scholz et al. to find decreased tolerance in *hangover* (*hang*) mutant flies, later confirmed in another study examining tolerance to EtOH-induced reduction of negative geotaxis [67,110]. *hang* encodes a nuclear zinc-finger protein that plays a role in cellular stress pathways, supporting the hypothesis that stress contributes to addiction phenotypes. Indeed, flies exposed to heat shock prior to naïve EtOH exposure display resistance to alcohol’s effects, indicating heat/EtOH cross-tolerance. In *hang* mutants, however, this cross-tolerance is largely abolished, suggesting that tolerance is mediated in part by *hang*-dependent cell stress pathways. Furthermore, mutation of either *hang* or *dunce* (*dnc*), a cAMP-degrading phosphodiesterase, produces similar tolerance deficits and reduced cellular stress responses [193]. The same group found that *hang* binds *dnc* mRNA, while *dnc* regulates *hang* function during tolerance formation. Thus, the effects of *hang* on EtOH tolerance may occur through cAMP-signaling-dependent stress response pathways. Based on initial findings with *hang*, Riley and colleagues performed a CGAS that revealed a significant association of the human ortholog *ZNF699* and alcohol dependence [109]. Human relevance was further shown by the finding of decreased *ZNF699* mRNA expression in the dorsolateral prefrontal cortex of postmortem tissue from individuals with an associated risk haplotype. Related to these pathways, Li et al. first investigated *jwa* (also known as *addicsin*; *ARL6IP5* in mammals) because of a similar association with stress responses [35]. Indeed, RNAi-mediated knockdown and overexpression in flies decreased and increased rapid EtOH tolerance, respectively. This gene exemplifies how, in contrast to unbiased screens, suspected AUD genes are often selected for further investigation because of known connections with previously implicated pathways or physiological processes in a one-gene-at-a-time approach. These higher-powered experiments increase the chances of finding moderate and small effect sizes, and their appeal as investigative or therapeutic targets is often bolstered by preexisting mechanistic hypotheses. Human studies then confirm translatability. In this case, Edenberg and colleagues independently performed human GWAS that supported an association between *ARL6IP5* and alcohol dependence, though no SNP reached genome-wide significance [34].

Forward screens have also been utilized to demonstrate that genes affecting responses to one drug of abuse are likely to affect other drug responses. Tsai et al. performed an unbiased screen for mutations affecting *Drosophila* cocaine sensitivity, which implicated the transcriptional repressor *dLmo* (*Bx*) [194]. Subsequent functional testing showed that *dLmo* loss increased EtOH sedation sensitivity, while overexpression decreased it [74]. Corroborating results from Sekhon and colleagues using the *Drosophila* Genetic Reference Panel (DGRP) found an association between *dLmo* and EtOH preference [52], and Kapoor et al. implicated the human ortholog *LMO1* in a GWAS looking at maximum drinks ever consumed within 24 h [39]. In mice, loss of orthologs *Lmo3* or *Lmo4* alters behavioral responses to cocaine, yet only *Lmo3* affects alcohol responses [74,195]. *dLmo* plays a role in both drug responses in flies, suggesting that evolutionary divergence has resulted in different mammalian homologs functioning in different pathways that are still integrated in flies (see also Reference [99]). Thus, translation of fly genetic discoveries into mammalian systems could benefit from accounting for this possibility by examining all mammalian orthologs of implicated fly genes. As another example of AUD gene discovery through testing of genes connected in pathways, Lasek and colleagues investigated anaplastic lymphoma kinase (*dAlk*) after microarray expression analyses revealed it to be negatively regulated by *dLmo* in flies [32]. *ALK* is involved in Erk signaling and other pathways [196]. Lasek et al. also found that *dAlk* fly mutants show increased resistance to EtOH sedation. A follow-up CGAS in the same study identified four human *ALK* polymorphisms linked to reduced EtOH responses. This gene was further validated in humans by a GWAS meta-analysis [33]. Overall, the initial screen of cocaine sensitivity by Tsai et al. facilitated discovery of various important AUD genes and biological principles, showing the promising potential of investigations into genes implicated in other substance use disorders.

Unbiased screens can become labor-intensive, so an alternative approach is to reduce screens to particular sets of candidate genes whose network or molecular roles have been previously implicated. Pinzon et al. used this approach to test effects of global histone demethylase (HDM) knockout on fly EtOH sedation sensitivity and tolerance [121]. Increasing evidence supports a role in AUDs of enzymes that modulate histone methylation and chromatin remodeling [180]. Since six out of seven phylogenetic families of human Jumonji C (JmjC) domain containing HDMs are represented by fly orthologs, each of the 13 known fly HDMs was knocked out and systematically tested for alcohol phenotypes. This study revealed effects of *KDM3, lid, NO66*, and *HSPBAP1*, the first three of which have orthologs that are upregulated in whole brains from alcohol-preferring mice [173]. Direct human evidence is lacking thus far, though the human ortholog of *NO66*, *RIOX1*, is downregulated in the amygdala of alcoholics [12]. The HDM study is exemplary for its success at performing a systematic screen of all genes within a family, which would be difficult to perform in higher model organisms. Nonetheless, an even more saturated screen of genes within the same pathways would be helpful for greater understanding of epistatic interactions [138].

In contrast to the structured gene discovery processes discussed thus far, AUD gene discovery and testing can also occur after independent convergence of results from multiple model systems. For instance, forward genetic transposon screens were the first to suggest a role of cAMP signaling in EtOH responses: Moore et al. found a sensitive mutant called *cheapdate* that was in fact an allele of *amnesiac* (*amn*), a known learning and memory gene thought to modulate adenylate cyclase [197]. Years later, Sekhon et al. independently implicated *amn* [52]. Tests of similar learning and memory genes revealed additional notable alcohol phenotypes caused by manipulations of *rutabaga (rut)* [11,142,197,198], encoding fly adenylyl cyclase, and *dnc* [193,199,200]. Separate from these pathways and studies, other studies have suggested alcohol-related roles of other genes in the network, including the cAMP-dependent protein kinase A (PKA) [114,197,201], protein kinase C (PKC) [79,202,203,204], CREB [205,206,207], and CREB binding protein (CBP) [117,208], consistently suggesting a causal role of cAMP signaling pathways in alcohol abuse. The K^+^ channel KCNQ is another example of the one-gene-at-a-time approach and the phenomenon of fly and human studies autonomously arriving at corroborating conclusions. KCNQ was examined because EtOH inhibits the non-inactivating K^+^ M current mediated by the channel, which normally reduces neural excitability [73]. KCNQ loss in flies augments sensitivity and tolerance to the sedating effects of ethanol [73]. This gene was again implicated in flies by GWAS and extreme QTL analyses using the DGRP resource and by RNAi knockdown [51]. Kendler et al. completed the picture by implicating human *KCNQ5* in a GWAS examining alcohol dependence, though again, no SNP achieved genome-wide significance [68]. Overall, whether as motivation or corroborating evidence for human investigations, biased and unbiased forward screens in *Drosophila* have and will continue to uncover many important genetic contributors to AUD. 

### 4.2. Fly GWAS and QTL Analyses

With almost five million known SNPs in the fly genome [9], sufficient genetic variation exists within *Drosophila* to allow effective gene discovery through GWAS and QTL studies. These relatively rare fly studies are valuable for their atypical yet comprehensive forward genetic approach. Although fly GWAS retain most of the advantages and disadvantages already discussed for human and rodent GWAS, they alleviate some problems by reducing environmental confounds and permitting quantification of phenotypic variability between individuals. Vast numbers of isogenic flies allow effective mapping of this variability to the genome, unlike in humans, where isogenic sample size is limited to sets of twins [209,210]. Further, linkage disequilibrium diminishes rapidly in flies compared to mammals, increasing the chances that SNPs associated with AUDs represent causal, rather than merely linked, variants [129,211]. Additionally, genetic tools available in *Drosophila* support GWAS and QTL success. The DGRP is a readily available stock collection comprised of over 200 lines created by extensive inbreeding of wild-caught females [129]. Each line has a sequenced genome, and many include transcriptome data [129,130]. Studies employing the DGRP can enhance results by advanced intercross mating schemes meant to amplify power and reveal effects of lower frequency alleles, as done by Fochler and colleagues [82]. Similar techniques were also employed to create the Drosophila Synthetic Population Resource (DSPR), including over 1600 recombinant inbred lines useful for mapping causative genetic variation [131]. Sekhon et al. used the DGRP to identify 507 genes associated with EtOH preference and 384 genes associated with both food and EtOH consumption [52]. Several fascinating studies by Morozova et al. have employed the DGRP for GWAS and extreme QTL analyses to corroborate AUD roles of genes like *Men* (see below), *dLmo*, and *rut* (see above) [11,51,79]. Using these techniques and transcriptomic approaches (discussed below), these studies also implicate whole gene networks, including those involved in dopamine synthesis and cAMP signaling, again showing high translatability. Finally, inbred fly lines are also useful for drawing associations between EtOH phenotypes, genetic variants, and/or expression profiles. For instance, Morozova et al. used microarrays to study the transcriptomes (discussed below) of fly lines bred for 35 generations for resistance or sensitivity [80]. After functional validation, they found that mutations in 32 out of 37 candidate genes indeed altered EtOH sensitivity. This high confirmation rate suggests that this method is effective for discovery of important genes mediating alcohol addiction.

### 4.3. Drosophila Transcriptomics

As an effective means of uncovering genes directly linked to alcohol intake, transcriptomics can be performed on flies that have been exposed to alcohol once or multiple times versus those that have not. As with human transcriptomics, these assays center on microarray and RNA-seq analyses. These approaches assume that genes differentially expressed in response to EtOH may be the same genes that contribute to AUD propensity and formation. Partially circumventing this assumption, researchers can enhance analysis with transcriptional comparison of controls and mutants known to affect alcohol responses. Genes found to display genotype × exposure interactions are especially likely to be involved in aberrant mutant phenotypes and possibly in wild-type responses. Thus, this suite of methods allows investigation into potential genetic mechanisms of both mutant phenotypes and AUD responses.

For instance, three independent studies performed similar microarray tests after EtOH exposure [63,81,160]. Synthesis of these results found that 14% of 1669 significantly dysregulated transcripts were identified in at least two of these studies, with 2% in all three [160]. These commonalities were relatively few in number, possibly due to different study designs or fly genetic backgrounds. However, their direction of change was remarkably consistent between studies, together suggesting highly robust gene associations that represent promising targets for future investigation. Indeed, many single genes were discovered, and later functionally validated, and further gene ontology analysis also revealed consistently altered genetic networks. These networks included many already implicated in mammals, such as those involved in metabolism, olfaction, epigenetics, and immunity. A notable gene identified in one of these microarray studies was *homer*, which is involved in post-synaptic regulation, especially of excitatory glutamatergic signaling [63]. *homer* transcripts decreased in response to EtOH exposure, and functional validation showed that *homer* is required for normal naïve EtOH sedation and tolerance. Although one CGAS found no association between human orthologs of *homer* and alcohol dependence [212], two unbiased studies suggested a role in human AUDs [60,61], and a large-scale GWAS implicated human *HOMER2* in reward-related learning and memory [62]. Given the connection between *homer* and NMDA receptors [63], these findings support the larger hypothesis of glutamatergic signaling being important in alcohol addiction.

As a further illustration of the effectiveness of transcriptomic approaches, Morozova et al. performed three unique studies testing fly transcriptomics in response to one or two EtOH exposures [11,80,81]. In an integrated approach, the 2011 study used unbiased screens to identify 139 unique mutations affecting EtOH sensitivity and tolerance [11]. Combining these hits with transcriptome data identified correlated transcriptional networks centered around nine genes whose mutation caused EtOH sensitivity and 12 mutations that caused resistance. A separate study in 2009 by the same group measured similar outputs but investigated associations between naïve EtOH sedation and mRNA profiles prior to sedation [79]. Many implicated genes in these studies were functionally validated in flies. Remarkably, all four studies identified malic enzyme (*Men*) as an important player in EtOH responses. Malic enzyme links glycolysis, the TCA cycle, and fatty acid synthesis. Alcoholics exhibit alcohol-induced fatty acid synthesis [213]. Using a CGAS to circumvent the multiple-testing problem of GWAS, the 2009 study found a significant association between human malic enzyme (*ME1*) and cocktail drinking, confirming the translatability of their *Drosophila* findings and demonstrating the effectiveness of using fly gene discovery to inform hypothesis-driven human association studies. Since then, Sekhon et al. corroborated the role of *Men* in flies by finding an association with EtOH preference [52], while Fochler et al. supported these findings with extreme QTL mapping and functional validation measuring fly alcohol consumption [82]. This *Men* narrative illustrates how a variety of transcriptomic approaches can effectively corroborate to elucidate the genetic underpinnings of AUDs.

One additional example of genes implicated using fly transcriptomics is worth noting: Ghezzi and colleagues measured expression of microRNAs (miRNAs) after EtOH exposure due to prior clues from rodents and flies of miRNA relevance in AUD [84]. miRNAs act as gene expression regulators by targeting specific mRNAs for degradation. Within 30 min of exposure, 14 miRNAs had altered expression. Of these, two out of seven tested were functionally validated: miR-6 and miR-310. Many of the putative targets of these miRNAs are established alcohol-related genes [138]. Human miR-92 is the sequence-related homolog of fly miR-310 and was shown to be upregulated in the prefrontal cortex of human alcoholics [83]. Thus, the usefulness of transcriptomics extends beyond protein-coding mRNAs.

### 4.4. Summary of Fly-to-Human Studies

Overall, *Drosophila* represent an effective and efficient model system, not just for gene validation and mechanistic investigations, but also for initial gene discovery. Behavioral screens, GWAS and QTL analyses, transcriptomics, and single-gene approaches each contribute unique insights into how genes and gene networks react to EtOH or prime the organism for altered responses that are potentially deleterious and predictive of AUD formation.

## 5. Future Directions

Flies have proven indispensable to the discovery and/or validation of numerous AUD-related genes. However, a quick scan of the literature summarizing these advancements, such as Park et al. [138], reveals that most confirmed AUD genes have simply been shown to affect alcohol responses, and much work remains to be done to uncover their mechanistic foundations. To date, most implicated genes have been studied only on a global level or sometimes on a neuronal level. This fact becomes problematic given that the roles of genes in AUD likely vary by brain region, cell population, and neuronal circuit (e.g., References [114] and [65], discussed above). Indeed, Ojelade et al. tested *Rsu1*, a known regulator of *Rac1*, and found that global *Rsu1* loss leads to high naïve EtOH preference, while *Rsu1* reduction in specific brain regions causes normal naïve preference but decreased learned preference [101]. Butts and colleagues similarly found an anatomy-specific role for Rac1 and cofilin [115]. In rodents, Rac1 in the dorsal versus ventral striatum plays opposite roles in cocaine-induced reward and spine maturation [214,215]. Furthermore, Scaplen et al. recently showed that population-level dopaminergic activation encodes alcohol rewards, whereas specific microcircuits encode cued activation of alcohol memories [216]. Ideally, mechanistic studies will employ fly genetic tools and increasing insight provided by the completed fly connectome [217] to parse out the specific cell populations or circuits in which genes play a given role. Moving forward, important sub-groups may include glial populations, which are generally under-researched.

Additionally, further investigation is warranted into genetic temporal underpinnings. Alcohol-related genes that cause developmental changes are useful for understanding AUD predisposition, but may not directly contribute to EtOH responses or AUD formation. Methods to manipulate genes only in adult *Drosophila* can rule out developmental influences, thereby more accurately modeling drinking problems and potential solutions for adult humans. Gene manipulation at different stages of alcohol exposures or stages of addiction is also warranted. Furthermore, most gene expression analyses only measure mRNA levels, which can show poor correlation with functional protein levels. Thus, proteomics should corroborate and supplement transcriptomic studies [218]. This approach has been utilized with flies and with human post-mortem tissue [219,220], but is generally not well explored. Moreover, there is great potential in using flies to screen potential drugs to treat AUDs, which has proven effective in other contexts [221,222]. Finally, many genes and pathways have been implicated in flies that, to our knowledge, lack studies examining their human correlates, despite obvious homology, probable shared pathways, and evidence of links to AUDs in mammals. These include *rut* (discussed above) [11,142,197,198], the dopamine/ecdysteroid receptor gene *DopEcR* [223,224], the deacetylase gene *Sirt1/Sir2* [78,81,160,225], various PKA genes [114,197,201], and *happyhour*, encoding a kinase that is a negative regulator of the (druggable) EGFR pathway mentioned above [158]. Further work is required to firmly establish the importance of some of these genes in *Drosophila* ethanol responses, whereas others are strongly implicated in flies but lack hypothesis-driven investigation in humans to advance the translational process.

## 6. Conclusions

Understanding the genetic bases of alcohol addiction is crucial for effective prevention and treatment. We have explained many effective methods to identify AUD-related genes using *Drosophila* as a starting or end point. For gene discovery, no single method far surpasses the other, so a variety of approaches should be used to maximize the chances of identifying critical genetic players and networks. Intersecting genes and pathways found using different methods and studies are strong candidates for further investigation and potential molecular targeting. In contrast, for qualitative filtering of potential AUD genes and for mechanistic understanding, *Drosophila* is an unparalleled model, given flies’ robust behavioral repertoire and convenient genetic toolkit. Indeed, conserved genetic targets that similarly influence alcohol responses despite the evolutionary distance separating these organisms are more likely to represent core elements of AUD propensity and development that have high therapeutic potential. Overall, *Drosophila* represents a powerful model to understand and mitigate human AUDs.

## Figures and Tables

**Table 1:** Genes implicated in both human and fly studies.

Function	Gene (Gray = Human, White = Fly)	Alcohol Phenotype	Citations
Receptor tyrosine kinase	*ALK*	LR; AD	[32,33]
	*dAlk*	SS	[32]
Cytoskeleton-associated transmembrane protein	*ARL6IP5*	AD	[34]
	*Jwa (addicsin)*	Rapid Tol (MET and Sed Rec)	[35]
Helix-loop-helix transcription factor	*ARNTL*	AC	[36]
	*ARNTL2*	AA	[36]
	*cyc*	Rapid Tol (SS)	[37]
Polycomb Repressor Complex 1 Modifier	*AUTS23*	AC; Max drinks; AC, post-mortem expression	[38,39,40]
	*tay*	SS	[40]
Chloride intracellular channel	*CLIC4*	Post-mortem expression	[41]
	*Clic*	eRING; SS	[42,43]
Dopamine beta-hydroxylase (norepinephrine synthesis)	*DBH*	AD; AD in women	[44,45]
	*Tbh*	Rapid Tol (MET)	[46]
		SS	[47]
		Olfactory preference	[48]
DOPA decarboxylase (dopamine and serotonin synthesis)	*DDC*	AC; Drug dependence	[49,50]
	*Ddc*	MET	[51]
		Correlation b/n expression and EtOH preference or intake	[52]
Metabotropic GABA receptor subunit	*GABBR1*	AD; AD	[53,54]
	*GABA-B-R1*	Sed Rec, Rapid Tol (Sed Rec)	[55]
Glutamate NMDA receptor subunit	*GRIN1*	AD; AD; AW seizure susceptibility	[56,57,58]
	*Nmdar1*	Sed Rec	[59]
Post-synaptic adaptor/regulator of glutamatergic synapses	*HOMER1*	AC; AC	[60,61]
	*HOMER2*	AC, alcohol-related problems; reward-related learning and memory	[60,62]
	*homer*	Exposure-induced expression, SS, Rapid Tol (SS)	[63]
Insulin-like growth factor receptor	*IGF1R*	LR	[64]
	*InR*	MET	[65]
		SS	[66]
Integrin beta subunit	*ITGB2*	LR	[64]
	*mys*	SS, Rapid Tol (SS)	[67]
Ca^2+^ and voltage-sensitive K^+^ channel	*KCNMA1*	AD; AD, early-onset AD	[34,68]
	*slo*	Rapid Tol (Sed Rec)	[69]
		Rapid Tol (SS), exposure-induced expression	[70]
		AW seizure susceptibility; AW seizure susceptibility	[71,72]
Voltage-gated K^+^ channel	*KCNQ5*	AD	[68]
	*KCNQ*	SS, Rapid Tol (SS)	[73]
		MET	[51]
LIM-type transcriptional regulator	*LMO1*	Max drinks	[39]
	*dLmo* (*Bx*)	SS	[74]
MADS-box transcription factor	*MEF2B*	SRE	[75]
	*MEF2C*	AC; AD	[76,77]
	*Mef2*	SS	[75]
		SS, Rapid Tol (SS)	[78]
Malic enzyme	*ME1*	Cocktail drinking	[79]
	*Men* (and paralogs)	Various	[11,79,80,81]
		Correlation b/n expression and EtOH preference or intake	[52]
		AC	[82]
Micro-RNA	*miR-92*	Post-mortem expression	[83]
	*miR-310*	Exposure-induced expression; Sed Rec	[84]
Cell adhesion molecule	*NCAM1*	AD; AD	[85,86]
	*Fas2*	MET	[87]
Neuropeptide Y	*NPY*	AD; AD; AD; AD; AD; AW	[36,88,89,90,91,92]
	*NPF*	SS	[93]
		Correlation b/n expression and EtOH preference or intake	[52]
Neuropeptide Y receptor	*NPY2R*	AD, AW, comorbid alcohol and cocaine dependence	[94]
	*NPFR*	SS	[93]
		Alcohol preference	[95]
		Correlation b/n expression and EtOH preference or intake	[52]
Transcriptional repressor involved in circadian rhythm	*PER2*	AC with sleep problems	[96]
	*PER3*	AA/AD	[97]
	*per*	Rapid Tol (Time to Sed)	[37]
		Circadian modulation of SS	[98]
Guanine exchange factor (GEF)	*PSD3*	AD, AC, adolescent binge drinking	[99]
	*Efa6*	Alcohol preference, SS, Rapid Tol (SS)	[99]
		SS	[100]
Ras suppressor	*RSU1*	AC	[101]
	*ics*	Alcohol preference	[101]
Ryanodine receptor	*RYR3*	AD, reward anticipation	[102]
	*RyR*	Rapid Tol (SS)	[102]
Vesicular monoamine transporter	*SLC18A1*	AUD, age at first alcohol use; AW	[103,104]
	*SLC18A2*	AD; AD	[105,106]
	*Vmat*	Correlation b/n expression and EtOH preference or intake	[52]
Norepinephrine transporter	*SLC6A2*	AD	[107]
	*DAT*	Act	[108]
Nuclear zinc-finger protein	*ZNF699*	AD, post-mortem expression	[109]
	*hang*	Rapid Tol (MET)	[110]
		Rapid Tol (eRING)	[42]

Columns show a brief description of the function of the gene product, the human (gray) or fly (white) orthologs, human or fly alcohol phenotypes associated with the gene variation, expression, or manipulation, with results from different studies separated by semi-colons and in respective order (Human: AA—alcohol abuse; AC—alcohol consumption (by volume or frequency); AD—alcohol dependence; AW—alcohol withdrawal; AUD—Alcohol use disorder diagnosis based on DSM IV criteria; LR—level of response to alcohol; Max drinks—most drinks consumed within a specified time period; post-mortem expression—transcript levels quantified from post-mortem tissue of alcoholics versus non-alcoholics; SRE—Self-Rating of the Effects of alcohol. Fly: Act—locomotor activity in the presence of alcohol; Alcohol preference—alcohol drinking/eating preference; eRING—ethanol Rapid Iterative Negative Geotaxis assay, measuring EtOH-induced reduction of negative geotaxis; exposure-induced expression—transcript levels quantified after exposure to EtOH versus mock exposure; MET—mean elution time from inebriometer; Olfactory preference—fraction of flies captured in a trap with alcohol odor vapor; Rapid Tol—rapid tolerance to the behavioral measure indicated in parentheses; Sed Rec—time required for flies to recover from sedation; SS—sensitivity to alcohol-induced sedation); and relevant citations.

## Abbreviations

ATAC-seq	Assay for transposase-accessible chromatin-sequencing
AUD	Alcohol use disorder
CGAS	Candidate gene association study
ChIP-seq	Chromatin immunoprecipitation-sequencing
DGRP	*Drosophila* Genetic Reference Panel
EGFR	Epidermal growth factor receptor
EtOH	Ethanol
FGFR	Fibroblast growth factor receptor
GWAS	Genome-wide association study
HDM	Histone demethylase
*Men*	Malic enzyme
miRNA	MicroRNA
PKA	Protein kinase A
QTL	Quantitative trait locus
RNA-seq	RNA-sequencing
UAS	Upstream activating sequence

## References

[1] USA Department of Health and Human Services, Substance Abuse and Mental Health Services Administration, Center for Behavioral Health Statistics and Quality (2018). National Survey on Drug Use and Health 2016 (NSDUH-2016-DS0001). https://datafiles.samhsa.gov/.

[2] Danaei G., Ding E.L., Mozaffarian D., Taylor B., Rehm J., Murray C.J., Ezzati M. (2009). The preventable causes of death in the United States: Comparative risk assessment of dietary, lifestyle, and metabolic risk factors. PLoS Med..

[3] W.H.O. (2014). Global Status Report on Alcohol and Health.

[4] Sacks J.J., Gonzales K.R., Bouchery E.E., Tomedi L.E., Brewer R.D. (2015). 2010 National and State Costs of Excessive Alcohol Consumption. Am. J. Prev. Med..

[5] American Psychiatric Association (2013). Diagnostic and Statistical Manual of Mental Disorders: DSM-5.

[6] Goldman D., Oroszi G., Ducci F. (2005). The genetics of addictions: Uncovering the genes. Nat. Rev. Genet..

[7] Nestler E.J. (2013). Cellular basis of memory for addiction. Dialogues Clin. Neurosci..

[8] Trudell J.R., Messing R.O., Mayfield J., Harris R.A. (2014). Alcohol dependence: Molecular and behavioral evidence. Trends Pharmacol. Sci..

[9] Engel G.L., Taber K., Vinton E., Crocker A.J. (2019). Studying alcohol use disorder using Drosophila melanogaster in the era of ‘Big Data’. Behav. Brain Funct. BBF.

[10] Farris S.P., Harris R.A., Ponomarev I. (2015). Epigenetic modulation of brain gene networks for cocaine and alcohol abuse. Front. Neurosci..

[11] Morozova T.V., Mackay T.F., Anholt R.R. (2011). Transcriptional networks for alcohol sensitivity in Drosophila melanogaster. Genetics.

[12] Ponomarev I., Wang S., Zhang L., Harris R.A., Mayfield R.D. (2012). Gene coexpression networks in human brain identify epigenetic modifications in alcohol dependence. J. Neurosci..

[13] Forero D.A., López-León S., Shin H.D., Park B.L., Kim D.-J. (2015). Meta-analysis of six genes (BDNF, DRD1, DRD3, DRD4, GRIN2B and MAOA) involved in neuroplasticity and the risk for alcohol dependence. Drug Alcohol Depend..

[14] Heath A.C., Whitfield J.B., Martin N.G., Pergadia M.L., Goate A.M., Lind P.A., McEvoy B.P., Schrage A.J., Grant J.D., Chou Y.-L. (2011). A Quantitative-Trait Genome-Wide Association Study of Alcoholism Risk in the Community: Findings and Implications. Biol. Psychiatry.

[15] Ioannidis J.P., Trikalinos T.A., Khoury M.J. (2006). Implications of small effect sizes of individual genetic variants on the design and interpretation of genetic association studies of complex diseases. Am. J. Epidemiol..

[16] Kim J.H., Park M., Yang S.Y., Jeong B.S., Yoo H.J., Kim J.-W., Chung J.-H., Kim S.A. (2006). Association study of polymorphisms in N-methyl-d-aspartate receptor 2B subunits (GRIN2B) gene with Korean alcoholism. Neurosci. Res..

[17] Edenberg H.J., Foroud T. (2013). Genetics and alcoholism. Nat. Rev. Gastroenterol. Hepatol..

[18] Edenberg H.J., Foroud T. (2014). Genetics of alcoholism. Handb. Clin. Neurol..

[19] Tawa E.A., Hall S.D., Lohoff F.W. (2016). Overview of the Genetics of Alcohol Use Disorder. Alcohol Alcohol. (Oxf. Oxfs.).

[20] Farris S.P., Arasappan D., Hunicke-Smith S., Harris R.A., Mayfield R.D. (2015). Transcriptome organization for chronic alcohol abuse in human brain. Mol. Psychiatry.

[21] Korpi E.R., den Hollander B., Farooq U., Vashchinkina E., Rajkumar R., Nutt D.J., Hyytiä P., Dawe G.S. (2015). Mechanisms of Action and Persistent Neuroplasticity by Drugs of Abuse. Pharmacol. Rev..

[22] Nestler E.J. (2001). Molecular basis of long-term plasticity underlying addiction. Nat. Rev. Neurosci..

[23] Warden A.S., Mayfield R.D. (2017). Gene expression profiling in the human alcoholic brain. Neuropharmacology.

[24] Deak J.D., Miller A.P., Gizer I.R. (2019). Genetics of alcohol use disorder: A review. Curr. Opin. Psychol..

[25] Hart A.B., Kranzler H.R. (2015). Alcohol Dependence Genetics: Lessons Learned From Genome-Wide Association Studies (GWAS) and Post-GWAS Analyses. Alcohol. Clin. Exp. Res..

[26] Salvatore J.E., Han S., Farris S.P., Mignogna K.M., Miles M.F., Agrawal A. (2019). Beyond genome-wide significance: Integrative approaches to the interpretation and extension of GWAS findings for alcohol use disorder. Addict. Biol..

[27] Rubin G.M., Yandell M.D., Wortman J.R., Gabor Miklos G.L., Nelson C.R., Hariharan I.K., Fortini M.E., Li P.W., Apweiler R., Fleischmann W. (2000). Comparative genomics of the eukaryotes. Science (N.Y.).

[28] Kaun K.R., Devineni A.V., Heberlein U. (2012). Drosophila melanogaster as a model to study drug addiction. Hum. Genet..

[29] Reiter L.T., Potocki L., Chien S., Gribskov M., Bier E. (2001). A systematic analysis of human disease-associated gene sequences in Drosophila melanogaster. Genome Res..

[30] Aquadro C.F., Bauer DuMont V., Reed F.A. (2001). Genome-wide variation in the human and fruitfly: A comparison. Curr. Opin. Genet. Dev..

[31] Shalaby N.A., Sayed R., Zhang Q., Scoggin S., Eliazer S., Rothenfluh A., Buszczak M. (2017). Systematic discovery of genetic modulation by Jumonji histone demethylases in Drosophila. Sci. Rep..

[32] Lasek A.W., Lim J., Kliethermes C.L., Berger K.H., Joslyn G., Brush G., Xue L., Robertson M., Moore M.S., Vranizan K. (2011). An evolutionary conserved role for anaplastic lymphoma kinase in behavioral responses to ethanol. PLoS ONE.

[33] Wang K.S., Liu X., Zhang Q., Pan Y., Aragam N., Zeng M. (2011). A meta-analysis of two genome-wide association studies identifies 3 new loci for alcohol dependence. J. Psychiatr. Res..

[34] Edenberg H.J., Koller D.L., Xuei X., Wetherill L., McClintick J.N., Almasy L., Bierut L.J., Bucholz K.K., Goate A., Aliev F. (2010). Genome-wide association study of alcohol dependence implicates a region on chromosome 11. Alcohol. Clin. Exp. Res..

[35] Li C., Zhao X., Cao X., Chu D., Chen J., Zhou J. (2008). The Drosophila homolog of jwa is required for ethanol tolerance. Alcohol Alcohol. (Oxf. Oxfs.).

[36] Kovanen L., Saarikoski S.T., Haukka J., Pirkola S., Aromaa A., Lönnqvist J., Partonen T. (2010). Circadian clock gene polymorphisms in alcohol use disorders and alcohol consumption. Alcohol Alcohol. (Oxf. Oxfs.).

[37] Pohl J.B., Ghezzi A., Lew L.K., Robles R.B., Cormack L., Atkinson N.S. (2013). Circadian genes differentially affect tolerance to ethanol in Drosophila. Alcohol. Clin. Exp. Res..

[38] Jorgenson E., Thai K.K., Hoffmann T.J., Sakoda L.C., Kvale M.N., Banda Y., Schaefer C., Risch N., Mertens J., Weisner C. (2017). Genetic contributors to variation in alcohol consumption vary by race/ethnicity in a large multi-ethnic genome-wide association study. Mol. Psychiatry.

[39] Kapoor M., Wang J.C., Wetherill L., Le N., Bertelsen S., Hinrichs A.L., Budde J., Agrawal A., Bucholz K., Dick D. (2013). A meta-analysis of two genome-wide association studies to identify novel loci for maximum number of alcoholic drinks. Hum. Genet..

[40] Schumann G., Coin L.J., Lourdusamy A., Charoen P., Berger K.H., Stacey D., Desrivieres S., Aliev F.A., Khan A.A., Amin N. (2011). Genome-wide association and genetic functional studies identify autism susceptibility candidate 2 gene (AUTS2) in the regulation of alcohol consumption. Proc. Natl. Acad. Sci. USA.

[41] Mayfield R.D., Lewohl J.M., Dodd P.R., Herlihy A., Liu J., Harris R.A. (2002). Patterns of gene expression are altered in the frontal and motor cortices of human alcoholics. J. Neurochem..

[42] Bhandari P., Hill J.S., Farris S.P., Costin B., Martin I., Chan C.L., Alaimo J.T., Bettinger J.C., Davies A.G., Miles M.F. (2012). Chloride intracellular channels modulate acute ethanol behaviors in Drosophila, Caenorhabditis elegans and mice. Genes Brain Behav..

[43] Chan R.F., Lewellyn L., DeLoyht J.M., Sennett K., Coffman S., Hewitt M., Bettinger J.C., Warrick J.M., Grotewiel M. (2014). Contrasting influences of Drosophila white/mini-white on ethanol sensitivity in two different behavioral assays. Alcohol. Clin. Exp. Res..

[44] Kohnke M.D., Kolb W., Kohnke A.M., Lutz U., Schick S., Batra A. (2006). DBH*444G/A polymorphism of the dopamine-beta-hydroxylase gene is associated with alcoholism but not with severe alcohol withdrawal symptoms. J. Neural Transm. (Vienna Austria 1996).

[45] Preuss U.W., Wurst F.M., Ridinger M., Rujescu D., Fehr C., Koller G., Bondy B., Wodarz N., Soyka M., Zill P. (2013). Association of functional DBH genetic variants with alcohol dependence risk and related depression and suicide attempt phenotypes: Results from a large multicenter association study. Drug Alcohol Depend..

[46] Scholz H., Ramond J., Singh C.M., Heberlein U. (2000). Functional ethanol tolerance in Drosophila. Neuron.

[47] Chen J., Wang Y., Zhang Y., Shen P. (2013). Mutations in Bacchus reveal a tyramine-dependent nuclear regulator for acute ethanol sensitivity in Drosophila. Neuropharmacology.

[48] Schneider A., Ruppert M., Hendrich O., Giang T., Ogueta M., Hampel S., Vollbach M., Büschges A., Scholz H. (2012). Neuronal basis of innate olfactory attraction to ethanol in Drosophila. PLoS ONE.

[49] Agrawal A., Lynskey M.T., Todorov A.A., Schrage A.J., Littlefield A.K., Grant J.D., Zhu Q., Nelson E.C., Madden P.A., Bucholz K.K. (2011). A candidate gene association study of alcohol consumption in young women. Alcohol. Clin. Exp. Res..

[50] Hack L.M., Kalsi G., Aliev F., Kuo P.H., Prescott C.A., Patterson D.G., Walsh D., Dick D.M., Riley B.P., Kendler K.S. (2011). Limited associations of dopamine system genes with alcohol dependence and related traits in the Irish Affected Sib Pair Study of Alcohol Dependence (IASPSAD). Alcohol. Clin. Exp. Res..

[51] Morozova T.V., Huang W., Pray V.A., Whitham T., Anholt R.R., Mackay T.F. (2015). Polymorphisms in early neurodevelopmental genes affect natural variation in alcohol sensitivity in adult drosophila. BMC Genom..

[52] Sekhon M.L., Lamina O., Hogan K.E., Kliethermes C.L. (2016). Common genes regulate food and ethanol intake in Drosophila. Alcohol (Fayettev. N. Y.).

[53] Kertes D.A., Kalsi G., Prescott C.A., Kuo P.H., Patterson D.G., Walsh D., Kendler K.S., Riley B.P. (2011). Neurotransmitter and neuromodulator genes associated with a history of depressive symptoms in individuals with alcohol dependence. Alcohol. Clin. Exp. Res..

[54] Reimers M.A., Riley B.P., Kalsi G., Kertes D.A., Kendler K.S. (2012). Pathway based analysis of genotypes in relation to alcohol dependence. Pharm. J..

[55] Dzitoyeva S., Dimitrijevic N., Manev H. (2003). Gamma-aminobutyric acid B receptor 1 mediates behavior-impairing actions of alcohol in Drosophila: Adult RNA interference and pharmacological evidence. Proc. Natl. Acad. Sci. USA.

[56] Wernicke C., Samochowiec J., Schmidt L.G., Winterer G., Smolka M., Kucharska-Mazur J., Horodnicki J., Gallinat J., Rommelspacher H. (2003). Polymorphisms in the N-methyl-D-aspartate receptor 1 and 2B subunits are associated with alcoholism-related traits. Biol. Psychiatry.

[57] Karpyak V.M., Geske J.R., Colby C.L., Mrazek D.A., Biernacka J.M. (2012). Genetic variability in the NMDA-dependent AMPA trafficking cascade is associated with alcohol dependence. Addict. Biol..

[58] Rujescu D., Soyka M., Dahmen N., Preuss U., Hartmann A.M., Giegling I., Koller G., Bondy B., Möller H.J., Szegedi A. (2005). GRIN1 locus may modify the susceptibility to seizures during alcohol withdrawal. Am. J. Med Genet. Part B Neuropsychiatr. Genet. Off. Publ. Int. Soc. Psychiatr. Genet..

[59] Troutwine B., Park A., Velez-Hernandez M.E., Lew L., Mihic S.J., Atkinson N.S. (2019). F654A and K558Q Mutations in NMDA Receptor 1 Affect Ethanol-Induced Behaviors in Drosophila. Alcohol. Clin. Exp. Res..

[60] Meyers J.L., Salling M.C., Almli L.M., Ratanatharathorn A., Uddin M., Galea S., Wildman D.E., Aiello A.E., Bradley B., Ressler K. (2015). Frequency of alcohol consumption in humans; the role of metabotropic glutamate receptors and downstream signaling pathways. Transl. Psychiatry.

[61] Heinrich A., Muller K.U., Banaschewski T., Barker G.J., Bokde A.L.W., Bromberg U., Buchel C., Conrod P., Fauth-Buhler M., Papadopoulos D. (2016). Prediction of alcohol drinking in adolescents: Personality-traits, behavior, brain responses, and genetic variations in the context of reward sensitivity. Biol. Psychol..

[62] Liu M., Jiang Y., Wedow R., Li Y., Brazel D.M., Chen F., Datta G., Davila-Velderrain J., McGuire D., Tian C. (2019). Association studies of up to 1.2 million individuals yield new insights into the genetic etiology of tobacco and alcohol use. Nat. Genet..

[63] Urizar N.L., Yang Z., Edenberg H.J., Davis R.L. (2007). Drosophila homer is required in a small set of neurons including the ellipsoid body for normal ethanol sensitivity and tolerance. J. Neurosci..

[64] Joslyn G., Ravindranathan A., Brush G., Schuckit M., White R.L. (2010). Human variation in alcohol response is influenced by variation in neuronal signaling genes. Alcohol. Clin. Exp. Res..

[65] Corl A.B., Rodan A.R., Heberlein U. (2005). Insulin signaling in the nervous system regulates ethanol intoxication in Drosophila melanogaster. Nat. Neurosci..

[66] Acevedo S.F., Peru y Colon de Portugal R.L., Gonzalez D.A., Rodan A.R., Rothenfluh A. (2015). S6 Kinase Reflects and Regulates Ethanol-Induced Sedation. J. Neurosci..

[67] Bhandari P., Kendler K.S., Bettinger J.C., Davies A.G., Grotewiel M. (2009). An assay for evoked locomotor behavior in Drosophila reveals a role for integrins in ethanol sensitivity and rapid ethanol tolerance. Alcohol. Clin. Exp. Res..

[68] Kendler K.S., Kalsi G., Holmans P.A., Sanders A.R., Aggen S.H., Dick D.M., Aliev F., Shi J., Levinson D.F., Gejman P.V. (2011). Genomewide association analysis of symptoms of alcohol dependence in the molecular genetics of schizophrenia (MGS2) control sample. Alcohol. Clin. Exp. Res..

[69] Cowmeadow R.B., Krishnan H.R., Atkinson N.S. (2005). The slowpoke gene is necessary for rapid ethanol tolerance in Drosophila. Alcohol. Clin. Exp. Res..

[70] Cowmeadow R.B., Krishnan H.R., Ghezzi A., Al’Hasan Y.M., Wang Y.Z., Atkinson N.S. (2006). Ethanol tolerance caused by slowpoke induction in Drosophila. Alcohol. Clin. Exp. Res..

[71] Ghezzi A., Krishnan H.R., Atkinson N.S. (2014). Susceptibility to ethanol withdrawal seizures is produced by BK channel gene expression. Addict. Biol..

[72] Ghezzi A., Pohl J.B., Wang Y., Atkinson N.S. (2010). BK channels play a counter-adaptive role in drug tolerance and dependence. Proc. Natl. Acad. Sci. USA.

[73] Cavaliere S., Gillespie J.M., Hodge J.J. (2012). KCNQ channels show conserved ethanol block and function in ethanol behaviour. PLoS ONE.

[74] Lasek A.W., Giorgetti F., Berger K.H., Tayor S., Heberlein U. (2011). Lmo genes regulate behavioral responses to ethanol in Drosophila melanogaster and the mouse. Alcohol. Clin. Exp. Res..

[75] Schmitt R.E., Shell B.C., Lee K.M., Shelton K.L., Mathies L.D., Edwards A.C., Grotewiel M. (2019). Convergent Evidence From Humans and Drosophila melanogaster Implicates the Transcription Factor MEF2B/Mef2 in Alcohol Sensitivity. Alcohol. Clin. Exp. Res..

[76] Evangelou E., Gao H., Chu C., Ntritsos G., Blakeley P., Butts A.R., Pazoki R., Suzuki H., Koskeridis F., Yiorkas A.M. (2019). New alcohol-related genes suggest shared genetic mechanisms with neuropsychiatric disorders. Nat. Hum. Behav..

[77] Muench C., Schwandt M., Jung J., Cortes C.R., Momenan R., Lohoff F.W. (2018). The major depressive disorder GWAS-supported variant rs10514299 in TMEM161B-MEF2C predicts putamen activation during reward processing in alcohol dependence. Transl. Psychiatry.

[78] Adhikari P., Orozco D., Randhawa H., Wolf F.W. (2019). Mef2 induction of the immediate early gene Hr38/Nr4a is terminated by Sirt1 to promote ethanol tolerance. Genes Brain Behav..

[79] Morozova T.V., Ayroles J.F., Jordan K.W., Duncan L.H., Carbone M.A., Lyman R.F., Stone E.A., Govindaraju D.R., Ellison R.C., Mackay T.F. (2009). Alcohol sensitivity in Drosophila: Translational potential of systems genetics. Genetics.

[80] Morozova T.V., Anholt R.R., Mackay T.F. (2007). Phenotypic and transcriptional response to selection for alcohol sensitivity in Drosophila melanogaster. Genome Biol..

[81] Morozova T.V., Anholt R.R., Mackay T.F. (2006). Transcriptional response to alcohol exposure in Drosophila melanogaster. Genome Biol..

[82] Fochler S., Morozova T.V., Davis M.R., Gearhart A.W., Huang W., Mackay T.F.C., Anholt R.R.H. (2017). Genetics of alcohol consumption in Drosophila melanogaster. Genes Brain Behav..

[83] Nunez Y.O., Mayfield R.D. (2012). Understanding Alcoholism Through microRNA Signatures in Brains of Human Alcoholics. Front. Genet..

[84] Ghezzi A., Zomeno M., Pietrzykowski A.Z., Atkinson N.S. (2016). Immediate-early alcohol-responsive miRNA expression in Drosophila. J. Neurogenet..

[85] Yang B.Z., Kranzler H.R., Zhao H., Gruen J.R., Luo X., Gelernter J. (2007). Association of haplotypic variants in DRD2, ANKK1, TTC12 and NCAM1 to alcohol dependence in independent case control and family samples. Hum. Mol. Genet..

[86] Yang B.Z., Kranzler H.R., Zhao H., Gruen J.R., Luo X., Gelernter J. (2008). Haplotypic variants in DRD2, ANKK1, TTC12, and NCAM1 are associated with comorbid alcohol and drug dependence. Alcohol. Clin. Exp. Res..

[87] Cheng Y., Endo K., Wu K., Rodan A.R., Heberlein U., Davis R.L. (2001). Drosophila fasciclinII is required for the formation of odor memories and for normal sensitivity to alcohol. Cell.

[88] Bhaskar L.V., Thangaraj K., Kumar K.P., Pardhasaradhi G., Singh L., Rao V.R. (2013). Association between neuropeptide Y gene polymorphisms and alcohol dependence: A case-control study in two independent populations. Eur. Addict. Res..

[89] Ilveskoski E., Kajander O.A., Lehtimäki T., Kunnas T., Karhunen P.J., Heinälä P., Virkkunen M., Alho H. (2001). Association of neuropeptide y polymorphism with the occurrence of type 1 and type 2 alcoholism. Alcohol. Clin. Exp. Res..

[90] Lappalainen J., Kranzler H.R., Malison R., Price L.H., Van Dyck C., Rosenheck R.A., Cramer J., Southwick S., Charney D., Krystal J. (2002). A functional neuropeptide Y Leu7Pro polymorphism associated with alcohol dependence in a large population sample from the United States. Arch. Gen. Psychiatry.

[91] Mottagui-Tabar S., Prince J.A., Wahlestedt C., Zhu G., Goldman D., Heilig M. (2005). A novel single nucleotide polymorphism of the neuropeptide Y (NPY) gene associated with alcohol dependence. Alcohol. Clin. Exp. Res..

[92] Okubo T., Harada S. (2001). Polymorphism of the neuropeptide Y gene: An association study with alcohol withdrawal. Alcohol. Clin. Exp. Res..

[93] Wen T., Parrish C.A., Xu D., Wu Q., Shen P. (2005). Drosophila neuropeptide F and its receptor, NPFR1, define a signaling pathway that acutely modulates alcohol sensitivity. Proc. Natl. Acad. Sci. USA.

[94] Wetherill L., Schuckit M.A., Hesselbrock V., Xuei X., Liang T., Dick D.M., Kramer J., Nurnberger J.I., Tischfield J.A., Porjesz B. (2008). Neuropeptide Y receptor genes are associated with alcohol dependence, alcohol withdrawal phenotypes, and cocaine dependence. Alcohol. Clin. Exp. Res..

[95] Shohat-Ophir G., Kaun K.R., Azanchi R., Mohammed H., Heberlein U. (2012). Sexual deprivation increases ethanol intake in Drosophila. Science (N.Y.).

[96] Comasco E., Nordquist N., Göktürk C., Aslund C., Hallman J., Oreland L., Nilsson K.W. (2010). The clock gene PER2 and sleep problems: Association with alcohol consumption among Swedish adolescents. Ups. J. Med. Sci..

[97] Banach E., Pawlak J., Kapelski P., Szczepankiewicz A., Rajewska-Rager A., Skibinska M., Czerski P., Twarowska-Hauser J., Dmitrzak-Weglarz M. (2018). Clock genes polymorphisms in male bipolar patients with comorbid alcohol abuse. J. Affect. Disord..

[98] van der Linde K., Lyons L.C. (2011). Circadian modulation of acute alcohol sensitivity but not acute tolerance in Drosophila. Chronobiol. Int..

[99] Gonzalez D.A., Jia T., Pinzon J.H., Acevedo S.F., Ojelade S.A., Xu B., Tay N., Desrivieres S., Hernandez J.L., Banaschewski T. (2018). The Arf6 activator Efa6/PSD3 confers regional specificity and modulates ethanol consumption in Drosophila and humans. Mol. Psychiatry.

[100] Peru Y.C.d.P.R.L., Acevedo S.F., Rodan A.R., Chang L.Y., Eaton B.A., Rothenfluh A. (2012). Adult neuronal Arf6 controls ethanol-induced behavior with Arfaptin downstream of Rac1 and RhoGAP18B. J. Neurosci..

[101] Ojelade S.A., Jia T., Rodan A.R., Chenyang T., Kadrmas J.L., Cattrell A., Ruggeri B., Charoen P., Lemaitre H., Banaschewski T. (2015). Rsu1 regulates ethanol consumption in Drosophila and humans. Proc. Natl. Acad. Sci. USA.

[102] Adkins A.E., Hack L.M., Bigdeli T.B., Williamson V.S., McMichael G.O., Mamdani M., Edwards A.C., Aliev F., Chan R.F., Bhandari P. (2017). Genomewide Association Study of Alcohol Dependence Identifies Risk Loci Altering Ethanol-Response Behaviors in Model Organisms. Alcohol. Clin. Exp. Res..

[103] Vaht M., Kiive E., Veidebaum T., Harro J. (2016). A Functional Vesicular Monoamine Transporter 1 (VMAT1) Gene Variant Is Associated with Affect and the Prevalence of Anxiety, Affective, and Alcohol Use Disorders in a Longitudinal Population-Representative Birth Cohort Study. Int. J. Neuropsychopharmacol..

[104] Dutta N., Helton S.G., Schwandt M., Zhu X., Momenan R., Lohoff F.W. (2016). Genetic Variation in the Vesicular Monoamine Transporter 1 (VMAT1/SLC18A1) Gene and Alcohol Withdrawal Severity. Alcohol. Clin. Exp. Res..

[105] Fehr C., Sommerlad D., Sander T., Anghelescu I., Dahmen N., Szegedi A., Mueller C., Zill P., Soyka M., Preuss U.W. (2013). Association of VMAT2 gene polymorphisms with alcohol dependence. J. Neural Transm. (Vienna Austria 1996).

[106] Schwab S.G., Franke P.E., Hoefgen B., Guttenthaler V., Lichtermann D., Trixler M., Knapp M., Maier W., Wildenauer D.B. (2005). Association of DNA polymorphisms in the synaptic vesicular amine transporter gene (SLC18A2) with alcohol and nicotine dependence. Neuropsychopharmacology.

[107] Clarke T.K., Dempster E., Docherty S.J., Desrivieres S., Lourdsamy A., Wodarz N., Ridinger M., Maier W., Rietschel M., Schumann G. (2012). Multiple polymorphisms in genes of the adrenergic stress system confer vulnerability to alcohol abuse. Addict. Biol..

[108] Kong E.C., Woo K., Li H., Lebestky T., Mayer N., Sniffen M.R., Heberlein U., Bainton R.J., Hirsh J., Wolf F.W. (2010). A pair of dopamine neurons target the D1-like dopamine receptor DopR in the central complex to promote ethanol-stimulated locomotion in Drosophila. PLoS ONE.

[109] Riley B.P., Kalsi G., Kuo P.H., Vladimirov V., Thiselton D.L., Vittum J., Wormley B., Grotewiel M.S., Patterson D.G., Sullivan P.F. (2006). Alcohol dependence is associated with the ZNF699 gene, a human locus related to Drosophila hangover, in the Irish Affected Sib Pair Study of Alcohol Dependence (IASPSAD) sample. Mol. Psychiatry.

[110] Scholz H., Franz M., Heberlein U. (2005). The hangover gene defines a stress pathway required for ethanol tolerance development. Nature.

[111] Rodan A.R., Rothenfluh A. (2010). The genetics of behavioral alcohol responses in Drosophila. Int. Rev. Neurobiol..

[112] Brand A.H., Perrimon N. (1993). Targeted gene expression as a means of altering cell fates and generating dominant phenotypes. Development.

[113] Jenett A., Rubin G.M., Ngo T.T., Shepherd D., Murphy C., Dionne H., Pfeiffer B.D., Cavallaro A., Hall D., Jeter J. (2012). A GAL4-driver line resource for Drosophila neurobiology. Cell Rep..

[114] Rodan A.R., Kiger J.A., Heberlein U. (2002). Functional dissection of neuroanatomical loci regulating ethanol sensitivity in Drosophila. J. Neurosci..

[115] Butts A.R., Ojelade S.A., Pronovost E.D., Seguin A., Merrill C.B., Rodan A.R., Rothenfluh A. (2019). Altered Actin Filament Dynamics in the Drosophila Mushroom Bodies Lead to Fast Acquisition of Alcohol Consumption Preference. J. Neurosci..

[116] Ghezzi A., Al-Hasan Y.M., Krishnan H.R., Wang Y., Atkinson N.S. (2013). Functional mapping of the neuronal substrates for drug tolerance in Drosophila. Behav. Genet..

[117] Ghezzi A., Krishnan H.R., Lew L., Prado F.J., Ong D.S., Atkinson N.S. (2013). Alcohol-induced histone acetylation reveals a gene network involved in alcohol tolerance. PLoS Genet..

[118] Lee K.M., Mathies L.D., Grotewiel M. (2019). Alcohol sedation in adult Drosophila is regulated by Cysteine proteinase-1 in cortex glia. Commun. Biol..

[119] Parkhurst S.J., Adhikari P., Navarrete J.S., Legendre A., Manansala M., Wolf F.W. (2018). Perineurial Barrier Glia Physically Respond to Alcohol in an Akap200-Dependent Manner to Promote Tolerance. Cell Rep..

[120] Petruccelli E., Feyder M., Ledru N., Jaques Y., Anderson E., Kaun K.R. (2018). Alcohol Activates Scabrous-Notch to Influence Associated Memories. Neuron.

[121] Pinzon J.H., Reed A.R., Shalaby N.A., Buszczak M., Rodan A.R., Rothenfluh A. (2017). Alcohol-Induced Behaviors Require a Subset of Drosophila JmjC-Domain Histone Demethylases in the Nervous System. Alcohol. Clin. Exp. Res..

[122] Luan H., Peabody N.C., Vinson C.R., White B.H. (2006). Refined spatial manipulation of neuronal function by combinatorial restriction of transgene expression. Neuron.

[123] Henry G.L., Davis F.P., Picard S., Eddy S.R. (2012). Cell type-specific genomics of Drosophila neurons. Nucleic Acids Res..

[124] Ma J., Weake V.M. (2014). Affinity-based isolation of tagged nuclei from Drosophila tissues for gene expression analysis. J. Vis. Exp..

[125] Thomas A., Lee P.J., Dalton J.E., Nomie K.J., Stoica L., Costa-Mattioli M., Chang P., Nuzhdin S., Arbeitman M.N., Dierick H.A. (2012). A versatile method for cell-specific profiling of translated mRNAs in Drosophila. PLoS ONE.

[126] Schauer T., Schwalie P.C., Handley A., Margulies C.E., Flicek P., Ladurner A.G. (2013). CAST-ChIP maps cell-type-specific chromatin states in the Drosophila central nervous system. Cell Rep..

[127] Kanca O., Bellen H.J., Schnorrer F. (2017). Gene Tagging Strategies To Assess Protein Expression, Localization, and Function in Drosophila. Genetics.

[128] Cusanovich D.A., Reddington J.P., Garfield D.A., Daza R.M., Aghamirzaie D., Marco-Ferreres R., Pliner H.A., Christiansen L., Qiu X., Steemers F.J. (2018). The cis-regulatory dynamics of embryonic development at single-cell resolution. Nature.

[129] Mackay T.F., Richards S., Stone E.A., Barbadilla A., Ayroles J.F., Zhu D., Casillas S., Han Y., Magwire M.M., Cridland J.M. (2012). The Drosophila melanogaster Genetic Reference Panel. Nature.

[130] Huang W., Massouras A., Inoue Y., Peiffer J., Ramia M., Tarone A.M., Turlapati L., Zichner T., Zhu D., Lyman R.F. (2014). Natural variation in genome architecture among 205 Drosophila melanogaster Genetic Reference Panel lines. Genome Res..

[131] King E.G., Merkes C.M., McNeil C.L., Hoofer S.R., Sen S., Broman K.W., Long A.D., Macdonald S.J. (2012). Genetic dissection of a model complex trait using the Drosophila Synthetic Population Resource. Genome Res..

[132] Mayfield R.D., Harris R.A., Schuckit M.A. (2008). Genetic factors influencing alcohol dependence. Br. J. Pharmacol..

[133] Morean M.E., Corbin W.R. (2010). Subjective response to alcohol: A critical review of the literature. Alcohol. Clin. Exp. Res..

[134] Ray L.A., Mackillop J., Monti P.M. (2010). Subjective responses to alcohol consumption as endophenotypes: Advancing behavioral genetics in etiological and treatment models of alcoholism. Subst. Use Misuse.

[135] Schuckit M.A. (1994). Low level of response to alcohol as a predictor of future alcoholism. Am. J. Psychiatry.

[136] Schuckit M.A. (2009). An overview of genetic influences in alcoholism. J. Subst. Abus. Treat..

[137] Narayanan A.S., Rothenfluh A. (2016). I Believe I Can Fly!: Use of Drosophila as a Model Organism in Neuropsychopharmacology Research. Neuropsychopharmacology.

[138] Park A., Ghezzi A., Wijesekera T.P., Atkinson N.S. (2017). Genetics and genomics of alcohol responses in Drosophila. Neuropharmacology.

[139] Robinson B.G., Atkinson N.S. (2013). Is alcoholism learned? Insights from the fruit fly. Curr. Opin. Neurobiol..

[140] Parr J., Large A., Wang X., Fowler S.C., Ratzlaff K.L., Ruden D.M. (2001). The inebri-actometer: A device for measuring the locomotor activity of Drosophila exposed to ethanol vapor. J. Neurosci. Methods.

[141] Singh C.M., Heberlein U. (2000). Genetic control of acute ethanol-induced behaviors in Drosophila. Alcohol. Clin. Exp. Res..

[142] Wolf F.W., Rodan A.R., Tsai L.T., Heberlein U. (2002). High-resolution analysis of ethanol-induced locomotor stimulation in Drosophila. J. Neurosci..

[143] Cohan F.M., Graf J.D. (1985). Latitudinal cline in drosophila melanogaster for knockdown resistance to ethanol fumes and for rates of response to selection for further resistance. Evolution.

[144] Berger K.H., Heberlein U., Moore M.S. (2004). Rapid and chronic: Two distinct forms of ethanol tolerance in Drosophila. Alcohol. Clin. Exp. Res..

[145] Krishnan H.R., Li X., Ghezzi A., Atkinson N.S. (2016). A DNA element in the slo gene modulates ethanol tolerance. Alcohol (Fayettev. N. Y.).

[146] Bayard M., McIntyre J., Hill K.R., Woodside J. (2004). Alcohol withdrawal syndrome. Am. Fam. Physician.

[147] Robinson B.G., Khurana S., Kuperman A., Atkinson N.S. (2012). Neural adaptation leads to cognitive ethanol dependence. Curr. Biol. CB.

[148] Kaun K.R., Azanchi R., Maung Z., Hirsh J., Heberlein U. (2011). A Drosophila model for alcohol reward. Nat. Neurosci..

[149] Devineni A.V., Heberlein U. (2009). Preferential ethanol consumption in Drosophila models features of addiction. Curr. Biol. CB.

[150] Peru Y.C.d.P.R.L., Ojelade S.A., Penninti P.S., Dove R.J., Nye M.J., Acevedo S.F., Lopez A., Rodan A.R., Rothenfluh A. (2014). Long-lasting, experience-dependent alcohol preference in Drosophila. Addict. Biol..

[151] Moskalev A., Zhikrivetskaya S., Krasnov G., Shaposhnikov M., Proshkina E., Borisoglebsky D., Danilov A., Peregudova D., Sharapova I., Dobrovolskaya E. (2015). A comparison of the transcriptome of Drosophila melanogaster in response to entomopathogenic fungus, ionizing radiation, starvation and cold shock. BMC Genom..

[152] Edenberg H.J. (2007). The genetics of alcohol metabolism: Role of alcohol dehydrogenase and aldehyde dehydrogenase variants. Alcohol Res. Health J. Natl. Inst. Alcohol Abus. Alcohol..

[153] Edenberg H.J., McClintick J.N. (2018). Alcohol Dehydrogenases, Aldehyde Dehydrogenases, and Alcohol Use Disorders: A Critical Review. Alcohol. Clin. Exp. Res..

[154] Gao Z., Lee P., Stafford J.M., von Schimmelmann M., Schaefer A., Reinberg D. (2014). An AUTS2-Polycomb complex activates gene expression in the CNS. Nature.

[155] Narita S., Nagahori K., Nishizawa D., Yoshihara E., Kawai A., Ikeda K., Iwahashi K. (2016). Association between AUTS2 haplotypes and alcohol dependence in a Japanese population. Acta Neuropsychiatr..

[156] Molnar C., de Celis J.F. (2013). Tay bridge is a negative regulator of EGFR signalling and interacts with Erk and Mkp3 in the Drosophila melanogaster wing. PLoS Genet..

[157] Schweitzer R., Shilo B.Z. (1997). A thousand and one roles for the Drosophila EGF receptor. Trends Genet..

[158] Corl A.B., Berger K.H., Ophir-Shohat G., Gesch J., Simms J.A., Bartlett S.E., Heberlein U. (2009). Happyhour, a Ste20 family kinase, implicates EGFR signaling in ethanol-induced behaviors. Cell.

[159] King I.F., Eddison M., Kaun K.R., Heberlein U. (2014). EGFR and FGFR pathways have distinct roles in Drosophila mushroom body development and ethanol-induced behavior. PLoS ONE.

[160] Kong E.C., Allouche L., Chapot P.A., Vranizan K., Moore M.S., Heberlein U., Wolf F.W. (2010). Ethanol-regulated genes that contribute to ethanol sensitivity and rapid tolerance in Drosophila. Alcohol. Clin. Exp. Res..

[161] Flavell S.W., Cowan C.W., Kim T.K., Greer P.L., Lin Y., Paradis S., Griffith E.C., Hu L.S., Chen C., Greenberg M.E. (2006). Activity-dependent regulation of MEF2 transcription factors suppresses excitatory synapse number. Science (N.Y.).

[162] Shalizi A., Gaudillière B., Yuan Z., Stegmüller J., Shirogane T., Ge Q., Tan Y., Schulman B., Harper J.W., Bonni A. (2006). A calcium-regulated MEF2 sumoylation switch controls postsynaptic differentiation. Science (N.Y.).

[163] Hawk J.D., Abel T. (2011). The role of NR4A transcription factors in memory formation. Brain Res. Bull..

[164] Ron D., Barak S. (2016). Molecular mechanisms underlying alcohol-drinking behaviours. Nat. Rev. Neurosci..

[165] Sivachenko A., Li Y., Abruzzi K.C., Rosbash M. (2013). The transcription factor Mef2 links the Drosophila core clock to Fas2, neuronal morphology, and circadian behavior. Neuron.

[166] Pulipparacharuvil S., Renthal W., Hale C.F., Taniguchi M., Xiao G., Kumar A., Russo S.J., Sikder D., Dewey C.M., Davis M.M. (2008). Cocaine regulates MEF2 to control synaptic and behavioral plasticity. Neuron.

[167] Contet C. (2012). Gene Expression Under the Influence: Transcriptional Profiling of Ethanol in the Brain. Curr. Psychopharmacol..

[168] Osterndorff-Kahanek E.A., Becker H.C., Lopez M.F., Farris S.P., Tiwari G.R., Nunez Y.O., Harris R.A., Mayfield R.D. (2015). Chronic ethanol exposure produces time- and brain region-dependent changes in gene coexpression networks. PLoS ONE.

[169] Zhou Z., Yuan Q., Mash D.C., Goldman D. (2011). Substance-specific and shared transcription and epigenetic changes in the human hippocampus chronically exposed to cocaine and alcohol. Proc. Natl. Acad. Sci. USA.

[170] Carr L.G., Foroud T., Bice P., Gobbett T., Ivashina J., Edenberg H., Lumeng L., Li T.K. (1998). A quantitative trait locus for alcohol consumption in selectively bred rat lines. Alcohol. Clin. Exp. Res..

[171] Crabbe J.C. (2008). Review. Neurogenetic studies of alcohol addiction. Philos. Trans. R. Soc. Lond. B Biol. Sci..

[172] Spence J.P., Liang T., Liu L., Johnson P.L., Foroud T., Carr L.G., Shekhar A. (2009). From QTL to candidate gene: A genetic approach to alcoholism research. Curr. Drug Abus. Rev..

[173] Mulligan M.K., Ponomarev I., Hitzemann R.J., Belknap J.K., Tabakoff B., Harris R.A., Crabbe J.C., Blednov Y.A., Grahame N.J., Phillips T.J. (2006). Toward understanding the genetics of alcohol drinking through transcriptome meta-analysis. Proc. Natl. Acad. Sci. USA.

[174] Robinson S.L., Thiele T.E. (2017). The Role of Neuropeptide Y (NPY) in Alcohol and Drug Abuse Disorders. Int. Rev. Neurobiol..

[175] Ehlers C.L., Li T.K., Lumeng L., Hwang B.H., Somes C., Jimenez P., Mathe A.A. (1998). Neuropeptide Y levels in ethanol-naive alcohol-preferring and nonpreferring rats and in Wistar rats after ethanol exposure. Alcohol. Clin. Exp. Res..

[176] Thiele T.E., Marsh D.J., Ste Marie L., Bernstein I.L., Palmiter R.D. (1998). Ethanol consumption and resistance are inversely related to neuropeptide Y levels. Nature.

[177] Shao L., Saver M., Chung P., Ren Q., Lee T., Kent C.F., Heberlein U. (2017). Dissection of the Drosophila neuropeptide F circuit using a high-throughput two-choice assay. Proc. Natl. Acad. Sci. USA.

[178] Grotewiel M., Bettinger J.C. (2015). Drosophila and Caenorhabditis elegans as Discovery Platforms for Genes Involved in Human Alcohol Use Disorder. Alcohol. Clin. Exp. Res..

[179] Roeder T., Seifert M., Kahler C., Gewecke M. (2003). Tyramine and octopamine: Antagonistic modulators of behavior and metabolism. Arch. Insect Biochem. Physiol..

[180] Ramirez-Roman M.E., Billini C.E., Ghezzi A. (2018). Epigenetic Mechanisms of Alcohol Neuroadaptation: Insights from Drosophila. J. Exp. Neurosci..

[181] Mulholland P.J. (2012). K(Ca)_2_ channels: Novel therapeutic targets for treating alcohol withdrawal and escalation of alcohol consumption. Alcohol (Fayettev. N.Y.).

[182] Schumann G., Liu C., O’Reilly P., Gao H., Song P., Xu B., Ruggeri B., Amin N., Jia T., Preis S. (2016). KLB is associated with alcohol drinking, and its gene product beta-Klotho is necessary for FGF21 regulation of alcohol preference. Proc. Natl. Acad. Sci. USA.

[183] Cope L.M., Munier E.C., Trucco E.M., Hardee J.E., Burmeister M., Zucker R.A., Heitzeg M.M. (2017). Effects of the serotonin transporter gene, sensitivity of response to alcohol, and parental monitoring on risk for problem alcohol use. Alcohol (Fayettev. N.Y.).

[184] Plemenitas A., Kastelic M., o Porcelli S., Serretti A., Dolžan V., Kores Plesnicar B. (2015). Alcohol Dependence and Genetic Variability in the Serotonin Pathway among Currently and Formerly Alcohol-Dependent Males. Neuropsychobiology.

[185] Seneviratne C., Franklin J., Beckett K., Ma J.Z., Ait-Daoud N., Payne T.J., Johnson B.A., Li M.D. (2013). Association, interaction, and replication analysis of genes encoding serotonin transporter and 5-HT3 receptor subunits A and B in alcohol dependence. Hum. Genet..

[186] Meltzer H., Marom E., Alyagor I., Mayseless O., Berkun V., Segal-Gilboa N., Unger T., Luginbuhl D., Schuldiner O. (2019). Tissue-specific (ts)CRISPR as an efficient strategy for in vivo screening in Drosophila. Nat. Commun..

[187] Port F., Strein C., Stricker M., Rauscher B., Heigwer F., Zhou J., Beyersdörffer C., Frei J., Hess A., Kern K. (2020). A large-scale resource for tissue-specific CRISPR mutagenesis in Drosophila. eLife.

[188] Rothenfluh A., Threlkeld R.J., Bainton R.J., Tsai L.T., Lasek A.W., Heberlein U. (2006). Distinct behavioral responses to ethanol are regulated by alternate RhoGAP18B isoforms. Cell.

[189] Ojelade S.A., Acevedo S.F., Kalahasti G., Rodan A.R., Rothenfluh A. (2015). RhoGAP18B Isoforms Act on Distinct Rho-Family GTPases and Regulate Behavioral Responses to Alcohol via Cofilin. PLoS ONE.

[190] Neasta J., Ben Hamida S., Yowell Q., Carnicella S., Ron D. (2010). Role for mammalian target of rapamycin complex 1 signaling in neuroadaptations underlying alcohol-related disorders. Proc. Natl. Acad. Sci. USA.

[191] Beckley J.T., Laguesse S., Phamluong K., Morisot N., Wegner S.A., Ron D. (2016). The First Alcohol Drink Triggers mTORC1-Dependent Synaptic Plasticity in Nucleus Accumbens Dopamine D1 Receptor Neurons. J. Neurosci..

[192] Cozzoli D.K., Kaufman M.N., Nipper M.A., Hashimoto J.G., Wiren K.M., Finn D.A. (2016). Functional regulation of PI3K-associated signaling in the accumbens by binge alcohol drinking in male but not female mice. Neuropharmacology.

[193] Ruppert M., Franz M., Saratsis A., Velo Escarcena L., Hendrich O., Gooi L.M., Schwenkert I., Klebes A., Scholz H. (2017). Hangover Links Nuclear RNA Signaling to cAMP Regulation via the Phosphodiesterase 4d Ortholog dunce. Cell Rep..

[194] Tsai L.T., Bainton R.J., Blau J., Heberlein U. (2004). Lmo mutants reveal a novel role for circadian pacemaker neurons in cocaine-induced behaviors. PLoS Biol..

[195] Lasek A.W., Kapfhamer D., Kharazia V., Gesch J., Giorgetti F., Heberlein U. (2010). Lmo4 in the nucleus accumbens regulates cocaine sensitivity. Genes Brain Behav..

[196] Gouzi J.Y., Moressis A., Walker J.A., Apostolopoulou A.A., Palmer R.H., Bernards A., Skoulakis E.M. (2011). The receptor tyrosine kinase Alk controls neurofibromin functions in Drosophila growth and learning. PLoS Genet..

[197] Moore M.S., DeZazzo J., Luk A.Y., Tully T., Singh C.M., Heberlein U. (1998). Ethanol intoxication in Drosophila: Genetic and pharmacological evidence for regulation by the cAMP signaling pathway. Cell.

[198] Xu S., Chan T., Shah V., Zhang S., Pletcher S.D., Roman G. (2012). The propensity for consuming ethanol in Drosophila requires rutabaga adenylyl cyclase expression within mushroom body neurons. Genes Brain Behav..

[199] Clarke T.K., Adams M.J., Davies G., Howard D.M., Hall L.S., Padmanabhan S., Murray A.D., Smith B.H., Campbell A., Hayward C. (2017). Genome-wide association study of alcohol consumption and genetic overlap with other health-related traits in UK Biobank (N = 112 117). Mol. Psychiatry.

[200] Peng Q., Bizon C., Gizer I.R., Wilhelmsen K.C., Ehlers C.L. (2019). Genetic loci for alcohol-related life events and substance-induced affective symptoms: Indexing the “dark side” of addiction. Transl. Psychiatry.

[201] Park S.K., Sedore S.A., Cronmiller C., Hirsh J. (2000). Type II cAMP-dependent protein kinase-deficient Drosophila are viable but show developmental, circadian, and drug response phenotypes. J. Biol. Chem..

[202] Chen J., Zhang Y., Shen P. (2008). A protein kinase C activity localized to neuropeptide Y-like neurons mediates ethanol intoxication in Drosophila melanogaster. Neuroscience.

[203] Chen J., Zhang Y., Shen P. (2010). Protein kinase C deficiency-induced alcohol insensitivity and underlying cellular targets in Drosophila. Neuroscience.

[204] Koyyada R., Latchooman N., Jonaitis J., Ayoub S.S., Corcoran O., Casalotti S.O. (2018). Naltrexone Reverses Ethanol Preference and Protein Kinase C Activation in Drosophila melanogaster. Front. Physiol..

[205] Wang Y., Ghezzi A., Yin J.C., Atkinson N.S. (2009). CREB regulation of BK channel gene expression underlies rapid drug tolerance. Genes Brain Behav..

[206] Wang Y., Krishnan H.R., Ghezzi A., Yin J.C., Atkinson N.S. (2007). Drug-induced epigenetic changes produce drug tolerance. PLoS Biol..

[207] Chen J., Hutchison K.E., Calhoun V.D., Claus E.D., Turner J.A., Sui J., Liu J. (2015). CREB-BDNF pathway influences alcohol cue-elicited activation in drinkers. Hum. Brain Mapp..

[208] Ghezzi A., Li X., Lew L.K., Wijesekera T.P., Atkinson N.S. (2017). Alcohol-Induced Neuroadaptation Is Orchestrated by the Histone Acetyltransferase CBP. Front. Mol. Neurosci..

[209] Morgante F., Sørensen P., Sorensen D.A., Maltecca C., Mackay T.F. (2015). Genetic Architecture of Micro-Environmental Plasticity in Drosophila melanogaster. Sci. Rep..

[210] Wu K.J., Kumar S., Serrano Negron Y.L., Harbison S.T. (2018). Genotype Influences Day-to-Day Variability in Sleep in Drosophila melanogaster. Sleep.

[211] Ober U., Ayroles J.F., Stone E.A., Richards S., Zhu D., Gibbs R.A., Stricker C., Gianola D., Schlather M., Mackay T.F. (2012). Using whole-genome sequence data to predict quantitative trait phenotypes in Drosophila melanogaster. PLoS Genet..

[212] Preuss U.W., Ridinger M., Rujescu D., Fehr C., Koller G., Wodarz N., Bondy B., Soyka M., Wong W.M., Zill P. (2010). No association of alcohol dependence with HOMER 1 and 2 genetic variants. Am. J. Med Genet. Part B Neuropsychiatr. Genet. Off. Publ. Int. Soc. Psychiatr. Genet..

[213] Lieber C.S. (2004). Alcoholic fatty liver: Its pathogenesis and mechanism of progression to inflammation and fibrosis. Alcohol (Fayettev. N.Y.).

[214] Li J., Zhang L., Chen Z., Xie M., Huang L., Xue J., Liu Y., Liu N., Guo F., Zheng Y. (2015). Cocaine activates Rac1 to control structural and behavioral plasticity in caudate putamen. Neurobiol. Dis..

[215] Dietz D.M., Sun H., Lobo M.K., Cahill M.E., Chadwick B., Gao V., Koo J.W., Mazei-Robison M.S., Dias C., Maze I. (2012). Rac1 is essential in cocaine-induced structural plasticity of nucleus accumbens neurons. Nat. Neurosci..

[216] Scaplen K.M., Talay M., Nunez K.M., Salamon S., Waterman A.G., Gang S., Song S.L., Barnea G., Kaun K.R. (2020). Circuits that encode and guide alcohol-associated preference. eLife.

[217] Xu C.S., Januszewski M., Lu Z., Takemura S.-y., Hayworth K.J., Huang G., Shinomiya K., Maitin-Shepard J., Ackerman D., Berg S. (2020). A Connectome of the Adult Drosophila Central Brain. BioRxiv.

[218] Huang W., Carbone M.A., Magwire M.M., Peiffer J.A., Lyman R.F., Stone E.A., Anholt R.R., Mackay T.F. (2015). Genetic basis of transcriptome diversity in Drosophila melanogaster. Proc. Natl. Acad. Sci. USA.

[219] Zhang Y., Shan B., Boyle M., Liu J., Liao L., Xu T., Yates J.R. (2014). Brain Proteome Changes Induced by Olfactory Learning in Drosophila. J. Proteome Res..

[220] Enculescu C., Kerr E.D., Yeo K.Y.B., Schenk G., Fortes M.R.S., Schulz B.L. (2019). Proteomics Reveals Profound Metabolic Changes in the Alcohol Use Disorder Brain. ACS Chem. Neurosci..

[221] Wang Y.Y., Ma W.W., Peng I.F. (2020). Screening of sleep assisting drug candidates with a Drosophila model. PLoS ONE.

[222] McBride S.M., Holloway S.L., Jongens T.A. (2012). Using Drosophila as a tool to identify Pharmacological Therapies for Fragile X Syndrome. Drug Discov. Today Technol..

[223] Aranda G.P., Hinojos S.J., Sabandal P.R., Evans P.D., Han K.A. (2017). Behavioral Sensitization to the Disinhibition Effect of Ethanol Requires the Dopamine/Ecdysone Receptor in Drosophila. Front. Syst. Neurosci..

[224] Petruccelli E., Li Q., Rao Y., Kitamoto T. (2016). The Unique Dopamine/Ecdysteroid Receptor Modulates Ethanol-Induced Sedation in Drosophila. J. Neurosci..

[225] Engel G.L., Marella S., Kaun K.R., Wu J., Adhikari P., Kong E.C., Wolf F.W. (2016). Sir2/Sirt1 Links Acute Inebriation to Presynaptic Changes and the Development of Alcohol Tolerance, Preference, and Reward. J. Neurosci..

